# A comprehensive lifestyle index and its associations with DNA methylation and type 2 diabetes among Ghanaian adults: the rodam study

**DOI:** 10.1186/s13148-024-01758-z

**Published:** 2024-10-16

**Authors:** C. A. Abidha, K. A. C. Meeks, F. P. Chilunga, A. Venema, R. Schindlmayr, C. Hayfron-Benjamin, Kerstin Klipstein-Grobusch, Frank P. Mockenhaupt, C. Agyemang, P. Henneman, I. Danquah

**Affiliations:** 1 Faculty of Medicine and University Hospital, Heidelberg Institute of Global Health (HIGH), Heidelberg University, Heidelberg, Germany; 2 Department of Public and Occupational Health, Amsterdam Public Health Research Institute, Amsterdam University Medical Centers, University of Amsterdam, Amsterdam, The Netherlands; 3 Center for Research on Genomics and Global Health, National Human Genome Research Institute, National Institutes of Health, Bethesda, USA; 4 Department of Human Genetics, Amsterdam Reproduction and Development Research Institute, Amsterdam University Medical Centers, University of Amsterdam, Amsterdam, The Netherlands; 5 Department of Physiology, University of Ghana Medical School, Accra, Ghana; 6 Department of Global Public Health and Bioethics, Julius Center for Health Sciences and Primary Care, Julius Global Health, University Medical Center Utrecht, Utrecht University, Utrecht, The Netherlands; 7 Division of Epidemiology and Biostatistics, School of Public Health, Faculty of Health Sciences, University of the Witwatersrand, Johannesburg, South Africa; 8 Institute of Tropical Medicine and International Health, Charité-Universitaetsmedizin Berlin, Corporate Member of Freie Universitaet Berlin and Humboldt-Universitaet Zu Berlin, and Berlin Institute of Health, Berlin, Germany; 9 Division of Endocrinology, Diabetes, and Metabolism, Department of Medicine, Johns Hopkins University School of Medicine, Baltimore, MD USA; 10 Department of Molecular Epidemiology, German Institute of Human Nutrition Potsdam-Rehbruecke (DIfE), Nuthetal, Germany

**Keywords:** Differentially methylated positions, DNA methylation (DNAm), Lifestyle index, Genome-wide association, Type 2 diabetes

## Abstract

**Background:**

A series of modifiable lifestyle factors, such as diet quality, physical activity, alcohol intake, and smoking, may drive the rising burden of type 2 diabetes (T2DM) among sub-Saharan Africans globally. It is unclear whether epigenetic changes play a mediatory role in the associations between these lifestyle factors and T2DM. We assessed the associations between a comprehensive lifestyle index, DNA methylation and T2DM among Ghanaian adults.

**Methods:**

We used whole-blood Illumina 450 k DNA methylation data from 713 Ghanaians from the Research on Obesity and Diabetes among African Migrants (RODAM) study. We constructed a comprehensive lifestyle index based on established cut-offs for diet quality, physical activity, alcohol intake, and smoking status. In the T2DM-free discovery cohort (*n* = 457), linear models were fitted to identify differentially methylated positions (DMPs) and differentially methylated regions (DMRs) associated with the lifestyle index after adjustment for age, sex, body mass index (BMI), and technical covariates. Associations between the identified DMPs and the primary outcome (T2DM), as well as secondary outcomes (fasting blood glucose (FBG) and HbA1c), were determined via logistic and linear regression models, respectively.

**Results:**

In the present study population (mean age: 52 ± 10 years; male: 42.6%), the comprehensive lifestyle index showed a significant association with one DMP annotated to an intergenic region on chromosome 7 (false discovery rate (FDR) = 0.024). Others were annotated to *ADCY7*, *SMARCE1*, *AHRR*, *LOXL2*, and *PTBP1* genes. One DMR was identified and annotated to the *GFPT2* gene (familywise error rate (FWER) from bumphunter bootstrap = 0.036). None of the DMPs showed significant associations with T2DM; directions of effect were positive for the DMP in the AHRR and inverse for all the other DMPs. Higher methylation of the ADCY7 DMP was associated with higher FBG (*p* = 0.024); LOXL2 DMP was associated with lower FBG (*p* = 0.023) and HbA1c (*p* = 0.049); and PTBP1 DMP was associated with lower HbA1c (*p* = 0.002).

**Conclusions:**

In this explorative epigenome-wide association study among Ghanaians, we identified one DMP and DMR associated with a comprehensive lifestyle index not previously associated with individual lifestyle factors. Based on our findings, we infer that lifestyle factors in combination, affect DNA methylation, thereby influencing the risk of T2DM among Ghanaian adults living in different contexts.

**Supplementary Information:**

The online version contains supplementary material available at 10.1186/s13148-024-01758-z.

## Introduction

Type 2 diabetes mellitus (T2DM) affects Sub-Saharan African (SSA) populations globally, but the actual burden differs according to geographic location [1]. Previous studies have reported an escalating prevalence of T2DM in SSA populations of similar genetic origin who live in Europe and the USA compared to their country of origin [2, 3].

Geographical differences in the prevalence of T2DM could be driven by a wide array of differences in the environment, including the built environment, such as neighbourhood walkability and the social environment, which may impact modifiable risk factors that influence lifestyle. Multiple studies have reported single modifiable risk factors associated with T2DM among SSA adults living in different geographic locations, including unhealthy dietary habits, smoking, physical inactivity, alcohol consumption, dyslipidaemia, blood pressure, and adiposity [4–11]. The exposure to these modifiable risk factors varies geographically and depends on the urbanization level [12]. Clearly, these factors interact and occur as part of combined lifestyle patterns [13]. A comprehensive lifestyle index would likely provide a better understanding of the combined effect of modifiable lifestyle factors on T2DM etiology as opposed to individual lifestyle factors. However, the combined effect of lifestyle patterns on T2DM in SSA populations and the underlying mechanisms have not been determined thus far.

One such underlying mechanism could be epigenetics. Epigenetics refer to the study of stable and heritable phenotype that arise from modifications in a chromosome devoid of alterations in the DNA sequence [14]. Epigenetic regulatory mechanisms are highly dynamic and may be affected by environmental factors [15–17]. DNA methylation is the most studied epigenetic mechanism. Epigenome-wide association studies (EWAS) have identified DNA methylation loci associated with T2DM in diverse populations [18–21] and with multiple individual lifestyle factors, such as smoking [22–24], physical activity [25], alcohol intake [26], and dietary patterns [27]. Since lifestyle factors can alter DNA methylation [16–18], DNA methylation could be one of the mechanisms mediating the association between modifiable risk factors and T2DM.

DNA methylation studies in sub-Saharan African (SSA) populations are limited. Research has demonstrated that DNA methylation changes can be population-specific, as shown in a South African study [28]. Previous work with Ghanaian adults identified several DNA methylation loci, including *TXNIP*, *C7orf50*, *CPT1A*, and *TPM4*, associated with T2DM [29]. *TXNIP*, which controls glucose uptake in cells by binding to GLUT1, has hypomethylation linked to smoking [30], and has shown differential methylation in T2DM and glycemic-related traits across various populations [31]. However, the drivers of these epigenetic changes remain unclear. Understanding the modifiable risk factors and their underlying mechanisms could help prevent the rising burden of T2DM in SSA populations.

In the present study, we aimed to explore the relationships between a comprehensive lifestyle index, DNA methylation and T2DM among adult Ghanaians living in different geographic contexts. The specific objectives were i) to operationalize unhealthy lifestyles through a comprehensive lifestyle index (CLI), ii) to identify DNA methylation loci associated with the lifestyle index, and iii) to explore the associations of the identified methylation loci with T2DM in a Ghanaian study population.

## Methods

### Study population

The present analysis uses cross-sectional baseline data from the Research on Obesity and Diabetes among African Migrants (RODAM) study. This was a multicentre study whose conceptual framework, design and data collection methods are described elsewhere [32, 33]. The RODAM study aimed to improve the understanding of the development of T2DM and obesity among African migrants at the phenotypic, genetic, and epigenetic levels. In brief, blood samples and data were collected from 6385 Ghanaian residents (aged ≥ 18 years) in five geographical locations, namely, rural Ghana, urban Ghana, the Netherlands, Germany, and the UK, between 2012 and 2015. Individuals were considered Ghanaians if they were born in Ghana and at least one parent was also born in Ghana or if they were born in Europe and both parents were born in Ghana. The methods of data collection were standardized across all study sites. Ethical approval was obtained from the ethics committees of the institutions involved in the four countries. All the study participants provided written informed consent.

### Sample selection

A subset of 736 participants from the RODAM study was selected for DNA methylation profiling (256 T2DM patients and 457 nondiabetic controls) (Supplementary Fig. 1). Of those, 713 passed quality control as described in detail elsewhere [29]. In the present study, we constructed a lifestyle index among these participants (*n* = 713). Furthermore, we used the T2DM-free control group (*n* = 426) as the discovery set for the associations between the lifestyle index and DNA methylation. The sample size was selected based on a genome-wide significance threshold with over 80% power to detect a 6% difference in methylation between the T2DM patients and controls [34].

## Data collection

### Assessment of sociodemographic, anthropometric and biomarker data

Information on demographics, socioeconomic status, and participants’ health history was obtained using self- or interviewer-administered questionnaires. Age (years), sex and study site were the main demographic information collected for the present study.

Well-trained study personnel conducted physical examinations, including anthropometric measurements, using validated devices and standardized procedures. Body weight (kg) was measured using the SECA 877 to the nearest 0.1 kg, and the portable stadiometer SECA 217 was used to measure height to the nearest 0.1 cm. Body mass index (BMI) was subsequently calculated in kg/m^2^, and waist circumference was measured in cm using a measuring tape.

T2DM status was considered as the primary outcome and defined based on self-reported diabetes status, current use of medication prescribed to treat T2DM, fasting blood glucose (FBG) ≥ 7 mmol/L or HbA1c ≥ 6.5%, according to the diagnostic criteria of the American Diabetes Association (ADA) [33]. FBG and HbA1c were classified as the secondary outcomes. FBG was measured in mmol/l using an ABX PENTRA 400 (Horiba ABX, Massachusetts, United States), whereas HbA1c was measured in mmol/mol by a high-performance liquid chromatography TOSOH G8 HPLC analyser (Tosoh Bioscience, Inc., South San Francisco, CA).

### Assessment of lifestyle factors

The lifestyle factors smoking status, physical activity, diet quality, and alcohol intake were selected for the construction of the lifestyle index. Smoking status was based on the answer to the guiding question, “Do you smoke?”. Participants responded by choosing from the following options: “yes”, “no, I have never smoked”, or “no, but I used to smoke”.

Additionally, the respondents were classified into three categories according to total physical activity using the International Physical Activity Questionnaire (IPAQ) [35]. The categories were based on the total physical activity in MET-minutes per week (MET = metabolic equivalent of task).

The categories were “high level”, “moderate level”, and “low level” of physical activity. Participants were assigned to the “high level” category if they reported vigorous-intensity activity for at least three days and had accumulated at least 1500 MET-minutes/week (OR) or seven or more days of any combination of walking, moderate-intensity or vigorous-intensity activities, accumulating at least 3000 MET-minutes/week. Those who had three or more days of vigorous activity of at least 20 min per day or 5 or more days of moderate-intensity activity and/or walking of at least 30 min per day or 5 or more days of any combination of walking, moderate-intensity or vigorous-intensity activities achieving a minimum of at least 600 MET-minutes/week were assigned to the “moderate level”. Finally, the participants who did not report any activity or who reported activities that were not enough to meet the high-level or moderate-level categories were assigned a “low-level” [35].

Information on dietary behaviour was collected using the Food Propensity Questionnaire (FPQ), which is tailor-made for the Ghanian population [36]. Energy intake and nutrient consumption were derived by translating the common household measures for portion sizes into grams per day and subsequently applying the energy and nutrient information from the West African Food Composition Table. Here, we also calculated individual-level diet quality according to the Diet Quality Index–International (DQI-I) [37, 38]. This sum score comprises 4 components, namely, variety, adequacy, moderation, and balance, and ranges from 0 to 100 points.

Alcohol intake was also calculated from the Ghana-FPQ data. Individuals were categorized into optimal or suboptimal intake groups according to the European Society of Cardiology (ESC) guidelines [39], which recommend a maximum daily intake of two units for men and one for women (1 unit of alcohol = 10 g of alcohol). Those who had never taken alcohol automatically fell into a third “no alcohol” group.

### DNA profiling and processing

DNA was extracted from whole-blood samples, after which methylation profiling was conducted by Source BioScience (Nottingham, UK). DNA was subjected to bisulfite treatment using an EZ DNA methylation Kit (Zymo Research, Orange, CA, USA) according to the manufacturer’s protocol. High-resolution melting analyses (from 65 to 98 °C) were also conducted to determine the quality of the conversion. The converted DNA was amplified and hybridized using an Illumina Human Methylation 450 K array. The raw 450 K data were subsequently processed for primary quality control using the MethylAid package (version 1.4.0) in “R” (version 3.2.2). The Minfi package (version 3.1.0.) was used for functional normalization. Finally, a sample size of 713 remained after quality control. Blood cell composition was estimated using a method similar to regression calibration as described by Houseman et al. [40]. The detailed procedure for DNA methylation profiling and processing are described elsewhere [29, 41].

### Statistical analysis

#### Handling missing data

The missing values were assumed to be either missing at random or completely at random. Multiple imputations were conducted for all the missing values using SAS and multiple imputation by chain equations (MICE) based on a regression model in “R” (version 3.2.2) (*n* = 10), with each prediction model depending on the variable type in focus (Supplementary Fig. 1).

#### Comprehensive lifestyle index (CLI)

The lifestyle index was calculated as an unweighted sum score based on current guidelines and cut-offs for each risk factor, i.e., diet quality, physical activity, alcohol intake, and smoking, as shown in Table 1. The scores were coded as “0”, “1”, or “2” for each of the risk factors. The lowest score was consequently allocated for the highest disease risk, and the highest score was allocated for the lowest disease risk. For example, for smoking and alcohol intake, the lowest score (“0”) was assigned to smokers and suboptimal alcohol users, whereas the highest score (“2”) was allocated to nonsmokers and abstainers. A medium score (“1”) was allocated to optimal alcohol users and former smokers. The DQI-I was divided into tertiles. The lowest score (“0”) was assigned to participants with ≥ 0 and ≤ 33.33, and the highest score (“2”) was assigned to those with > 66.66 and ≤ 100. Participants with scores > 33.33 and ≤ 66.66 were allocated a score of “1”. For physical activity, those who had a “high level” were allocated the highest score points, i.e., (“2”), whereas those who had a “low level” scored the lowest, i.e., (“0”). Participants who had a “moderate level” were assigned a score of (“1”).

**Table 1:** Allocation of scores to the modifiable risk factors for T2DM The highest score is allocated to the healthiest lifestyle (lowest risk for noncommunicable diseases)

Lifestyle factor	Categories	Points
Smoking	Current smokers	0
	Former smokers	1
	Never smokers	2
Alcohol intake	Alcohol units/day > 1♀ or > 2♂	0
	Alcohol units/day > 0 & < = 1♀ or < = 2♂	1
	Abstainers Alcohol units/day = 0	2
Physical activity	Low level	0
	Moderate level	1
	High level	2
Diet	DQII tertile 1	0
	DQII tertile 2	1
	DQII tertile 3	2

Alcohol intake was categorized according to the ESC guidelines, and physical activity was categorized according to the IPAQ guidelines

*ESC:* European Society of Cardiology

*DQII:* Diet Quality Index International

*IPAQ:* International Physical Activity Questionnaire

After that, the total scores were calculated across all the risk factors for each participant. A total score ≥ 0 or ≤ 4 indicated a high risk. Conversely, if the total score was > 4 or ≤ 8, the risk level was termed “low risk”. These risk levels were ultimately coded into binary variables to form an index. Suppose the risk level is “high risk”, then the risk factor index = “1”; if the risk level is “low risk”, then the risk factor index = “0”. This variable was then subjected to subsequent regression analyses.

Finally, a correlation matrix was generated to investigate the correlation between the lifestyle index and individual scores of the lifestyle factors using the corrplot() function in “R” (version 3.2.2). BMI was also included in the matrix.

#### Descriptive statistics

A descriptive summary of the study population, including demographic variables, socioeconomic status, anthropometric data, estimated cell distributions, and the selected lifestyle risk factors across T2DM status and geographical location, was conducted for the total study population (*n* = 713) using the “Table 1” package in “R” (version 4.3.1). Continuous variables are presented as medians and interquartile ranges. Age is presented as the mean and standard deviation. The proportions of males are presented as percentages (*n*). All the other categorical variables are presented as percentages.

#### Identification of differentially methylated positions (DMPs)

**Fig. 1:**
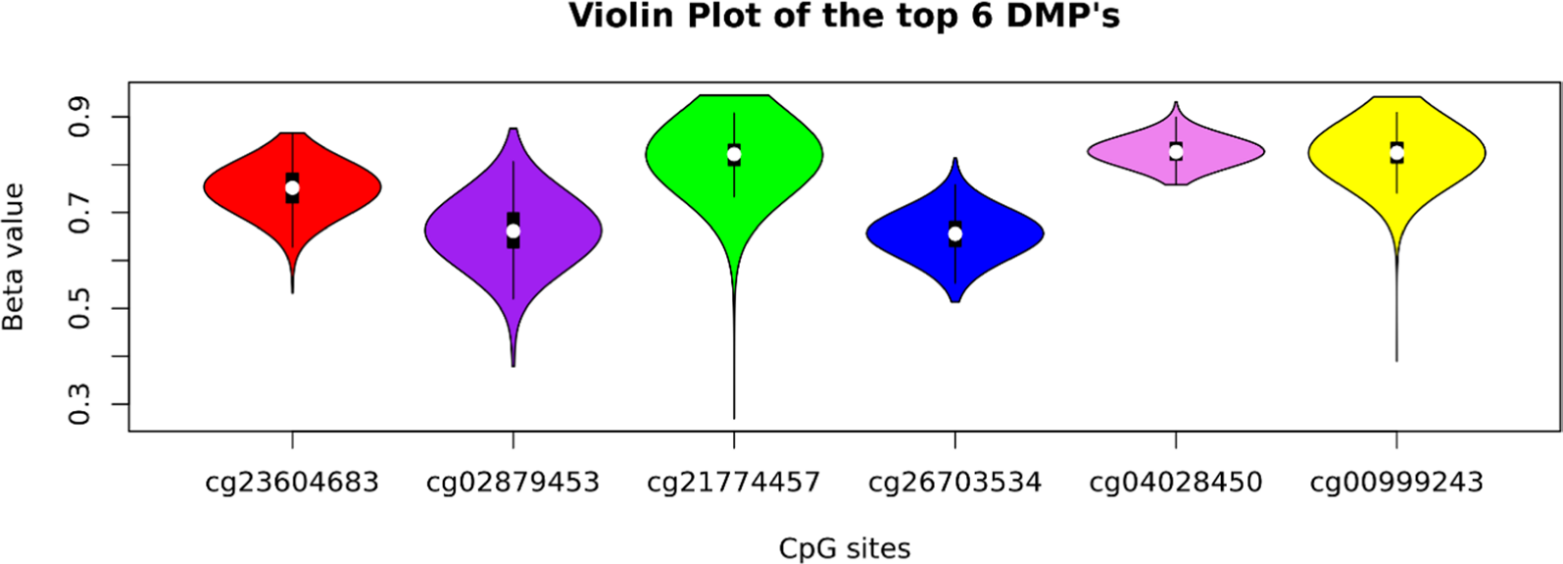
Violin plot showing the percentage beta value distribution for the top 6 DMPs. The plot examines the effect of single nucleotide polymorphisms driving observed DNA methylation variation. This finding confirmed that the methylation sites are not driven by single nucleotide polymorphisms (SNPs)

Linear regression analyses were conducted using the LmFit function of the Limma package (v3.58.1; [42]) to identify differentially methylated positions (DMPs) associated with high and low adherence to the lifestyle index among T2DM controls (*n* = 426), taking the lifestyle index as the exposure variable, and the DMPs as the outcome variables. The model was adjusted for age, sex, study site and technical covariates including estimated cell counts, hybridization batch and array position. A secondary model was additionally adjusted for BMI. The bacon package (v1.4.0; [43]) was utilized to address possible inflation of our test statistics, potentially caused by systematic biases. The final model fit was assessed using QQ and violin plots (Supplementary Figs. 2a–d and 3a, b). False discovery rate (FDR)-adjusted bacon p values were calculated using the Benjamini–Hochberg method to correct for multiple testing. An FDR-adjusted *p* value < 0.05 was considered to indicate genome-wide significance. A violin plot showing the percentage beta value distribution for the top DMPs was generated to rule out the effect of single nucleotide polymorphisms driving observed DNA methylation variation (Fig. 1).

#### Identification of differentially methylated regions (DMRs)

To identify differentially methylated regions (DMRs), the bumphunter function of the Bioconductor package minfi (v3.1.0.; [44] was utilized [45]. A DMR was defined as 3 or more CpG sites within the cluster. A DMR with a familywise error rate (FWER) < 0.05 was considered genome-wide significant. Optimization was performed using a 0.01 cut-off, corresponding to a 1.0% difference in beta values, while bootstrapping was performed at 500 permutations.

#### Associations of DMPs and DMRs with T2DM and markers of glucose metabolism

Next, we determined the associations between a 1% increase in the DNA methylation of the top identified DMPs and T2DM via two logistic regression models. No adjustments were made in the base model, whereas age, sex, geographical location, and BMI were controlled for in the secondary model. In addition, the associations between the top DMPs and biomarkers of T2DM, including FBG and HbA1c, were determined via linear regression analyses. Furthermore, we determined the association between the identified DMR and T2DM, and biomarkers of T2DM i.e., HbA1c and FBG.

### Post hoc analyses

#### Enrichment analysis and annotation

To annotate, understand and interpret the identified DMPs and DMRs, the EWAS catalog (http://www.ewascatalog.org) [46], the GeneCards database (http://www.genecards.org/), the EWAS Atlas (EWAS Open Platform (cncb.ac.cn) [46], and the GWAS catalog (https://www.ebi.ac.uk/gwas/) were queried. To visualize the identified DMPs, their specific gene annotations, the genes surrounding them, their expression patterns, the presence of histone modifications and their preservation across species, the UCSC Genome browser was used. Furthermore, to determine whether the identified CpGs are enriched in DNAse I hypersensitivity site hotspots in specific tissue types, we utilized the eFORGE tool (http://eforge.cs.ucl.ac.uk/).

To determine the presence of SNPs or any associations between SNPs and the identified DMPs, we utilized the mQTL database (http://www.mqtldb.org/). mQTL SNPs were subsequently evaluated in the dbSNP database (https://www.ncbi.nlm.nih.gov/snp/). The EWAS Atlas was additionally used for enrichment analyses and annotation using an enrichment tool kit (EWAS Open Platform (cncb.ac.cn)) [86]. The top 100 CpG sites were used as the input, yielding an output that was categorized into 5 enrichment and annotation sections.

#### Mediation analysis

A standard mediation analysis for the top DMPs was conducted using the mediation package (v4.5.0; [47] in R. Here, we aimed to investigate the hypothesis that there is an indirect effect of the lifestyle index on the likelihood of a person developing T2DM via modification through DNA methylation. The mean beta values for the top DMPs were used in this analysis. First, we tested the total effect by testing whether any change in the lifestyle index impacts T2DM. Second, we tested the effect of the lifestyle index on DNA methylation. Third, we tested the effect of the lifestyle index and DNA methylation on T2DM simultaneously. Finally, we estimated the various quantities for causal mediation analysis by comparing the direct and indirect effects to clarify what is going on in the data. The significance of the indirect effect was tested by bootstrapping 1000 times at a 95% confidence interval (-0.01 to 0.02).

## Results

### Descriptive statistics

Of the total analytical sample, 304 were males, whereas 409 were females (Table 2). The mean age of the study participants was 51 years. A total of 40.3% of the participants had been diagnosed with T2DM, whereas 59.7% were not diagnosed with T2DM. Approximately half of the participants (51.3%) resided in Europe, majority being first generation migrants. The mean FBG was 6.4 ± 3.3 mmol/L, whereas the mean HbA1c was 46 mmol/mol. The mean body weight was 73 ± 16 kg, and the mean BMI was 27 ± 5.5 kg/m^2^.

**Table 2:** Characteristics of the Study Population by T2DM status and geographical location

	Total (*N* = 713)	T2DM cases (*N* = 287)	T2DM controls (*N* = 426)	Rural Ghana (*N* = 104)	Urban Ghana (*N* = 243)	Europe (*N* = 366)
Gender (%male)	42.6	46.3	40.1	30.8	29.6	54.6
Age (Years)	51 ± 9.9	52 ± 10	51 ± 9.4	56 ± 8.9	51 ± 9.8	50 ± 9.7
T2DM status						
Cases	287(40.3%)	NA	NA	42(40.4%)	94(38.7%)	151(41.3)
Controls	426(59.7%)	NA	NA	62(59.6%)	149(61.3%)	215 (58.7)
*Lifestyle risk factors*						
Alcohol intake						
Abstainer	439 (61.6%)	172 (59.9%)	267 (62.7%)	55(52.9%)	171(70.4%)	213(58.2)
Heavy drinker	21 (2.9%)	6 (2.1%)	15 (3.5%)	2(1.9%)	1(0.4%)	18(4.9)
Moderate drinker	253 (35.5%)	109 (38.0%)	144 (33.8%)	47(45.2%)	71(29.2%)	135(36.9)
Smoking						
Former smoker	65 (9.1%)	30 (10.5%)	35 (8.2%)	11(10.6%)	22(9.1%)	32(8.7)
Non smoker	632 (88.6%)	251 (87.5%)	381 (89.4%)	93(89.4%)	220(90.5%)	319(87.2)
Smoker	16 (2.2%)	6 (2.1%)	10 (2.3%)	0(0%)	1(0.4%)	15(4.1)
Physical activity						
High level	297 (41.7%)	102 (35.5%)	195 (45.8%)	49(47.1%)	101(41.6%)	147(40.2)
Low level	253 (35.5%)	111 (38.7%)	142 (33.3%)	31(29.8%)	98(40.3%)	124(33.9)
Moderate level	163 (22.9%)	74 (25.8%)	89 (20.9%)	24(23.1%	44(18.1%)	95(26.0)
DQII	56 [37, 76]	56 [37, 71]	56 [40, 76]	55 [40, 76]	55 [40, 71]	57 [37, 72]
CLI						
High risk	183 (25.7%)	82 (28.6%)	101 (23.7%)	29(27.9%)	53(21.8%)	101(27.6)
Low risk	530 (74.3%)	205 (71.4%)	325 (76.3%)	75(72.1%)	190(78.2%)	265(72.4)
HbA1c (mmol/mol)	38 [12, 150]	53 [23, 150]	36 [12, 150]	33 [12, 150]	38 [15, 150]	39 [20, 150]
FBG (mmol/L)	5.2 [3.1, 35]	7.4 [3.6, 35]	4.9 [3.1, 7.0]	5.3 [3.4, 35]	5.2 [3.1, 30]	5.1 [3.4, 18]
Waist (cm)	90 [55, 140]	95 [55, 140]	86 [65, 130]	82 [65, 110]	88 [65, 140]	95 [55, 130]
BMI	26 [16, 52]	28 [16, 52]	25 [16, 44]	22 [16, 33]	25 [16, 52]	28 [19, 45]

Continuous data are presented as medians and interquartile ranges. Age is presented as the mean and standard deviation. The proportions of males are presented as percentages (*n*). All the other categorical variables are presented as percentages

*T2DM*:  type 2 diabetes mellitus

*DQII*: Diet Quality Index International

*CLI:* comprehensive lifestyle index

*FBG:* fasting blood glucose

**Fig. 2:**
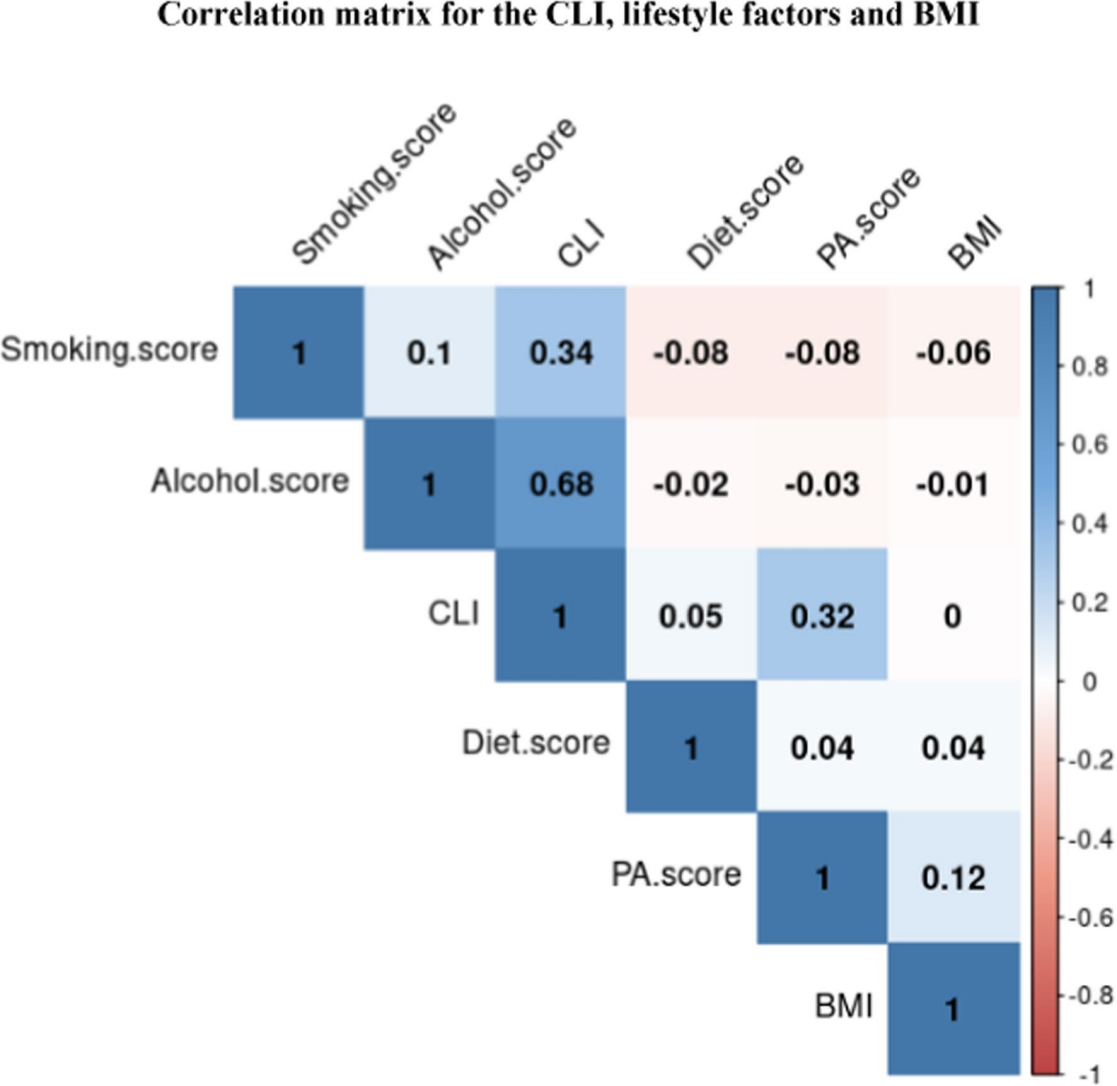
The figure shows the dependence between the CLI and the individual scores of the components of the CLI and BMI. Positive correlations are displayed in blue, and negative correlations are displayed in red. The colour intensity is proportional to the correlation coefficient. On the right side of the correlogram, the legend colour shows the correlation coefficients and the corresponding colours. Correlations with a *p* value > 0.01 were considered insignificant. *CLI* comprehensive lifestyle index, *PA.score* physical activity score

For the lifestyle index, most of the participants were in the low-risk group (74.3%), with a majority being nonsmokers (88.6%) and nonconsumers of alcohol (61.6%) (Table 2). The average DQII was 56 ± 6.1, while physical activity levels were equally distributed, with 41.7% and 35.5% of the participants in the high- and low-level categories, respectively. Accordingly, the correlation matrix showed that variation in the lifestyle index scores was mostly driven by alcohol consumption (r^2^ = 0.68), followed by smoking (*r*^2^ = 0.34) and physical activity (*r*^2^ = 0.32) (Fig. 2). The correlations between the lifestyle index and diet and BMI were not statistically significant. According to the association analyses between individual lifestyle risk factors and T2DM, physical activity was significantly associated with T2DM (*p* = 0.01) and FBG (*p* = 0.031).

#### Differentially methylated positions (DMPs) associated with the comprehensive lifestyle index

**Fig. 3:**
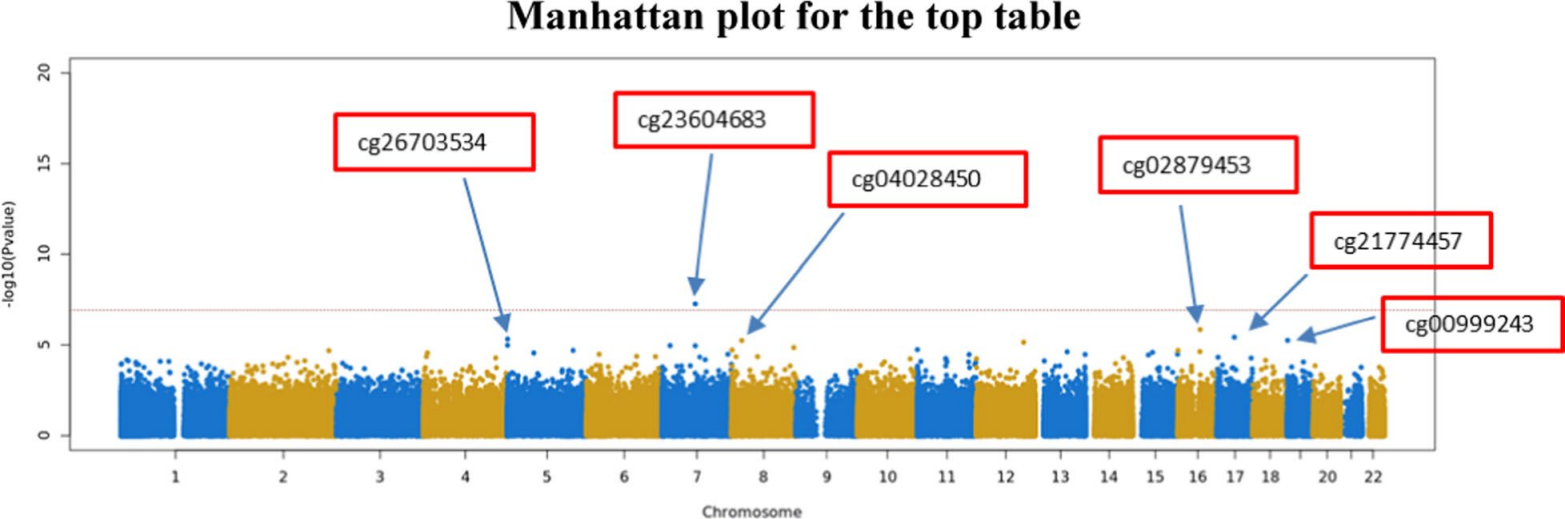
*M*-value top table Manhattan plot showing the top 6 DMPs. The DMPs are highlighted in the red boxes. The *x* axis is the chromosomal position, and the *y* axis is the significance on a − log10 scale. The arrows point to the exact position of each DMP on the chromosome. The horizontal line represents the genome-wide significance level (FDR < 0.05). The *p* values were corrected for genomic control

We identified one genome-wide significant DMP annotated to an intergenic region on chromosome 7 (cg23604683; FDR = 0.024) (Fig. 3, Table 3, and Supplementary Fig. 3). The five DMPs with the lowest nominal p values additionally included DMPs annotated to *ADCY7* (cg02879453), *SMARCE1* (cg21774457), *AHRR* (cg26703534), and *LOXL2* (cg04028450) (Table 3). These top five largely overlapped between the models with and without adjustment for BMI. There was only one DMP within the top five with the lowest nominal p values in the BMI-adjusted model; this DMP was not included in the top five in the base model and was annotated to *PTBP1* (cg00999243). Five of these six DMPs were hypomethylated in patients with a high lifestyle index compared to those with a low lifestyle index, and only one DMP was hypermethylated, with effect sizes of approximately 1–2% (Table 3). Notably, the identified CpGs were not DNAse I hypersensitivity site hotspots.

**Table 3:** Differentially methylated positions associated with a comprehensive lifestyle index among Ghanaian adults without T2DM (*n* = 426)

CpG	Chr	Position	Gene	Feature	Delta *ß* value	Base model		Adjusted for BMI	
						*P*-value	FDR	*P*-value	FDR
cg23604683	chr7	75,779,470	*Intergenic region*		− 0.0249	5.71E−08	0.0245	5.24E−08	0.0225
cg02879453	chr16	50,321,818	*ADCY7*	TSS200	− 0.026	1.50E−06	0.3228	1.43E−06	0.306
cg21774457	chr17	38,800,072	*SMARCE1*	Body	0.0107	4.51E−06	0.4248	3.76E−06	0.4079
cg26703534	chr5	377,358	*AHRR*	Body	− 0.0191	4.89E−06	0.4248	4.75E−06	0.4079
cg04028450	chr8	23,201,488	*LOXL2*	Body	− 0.0115	5.86E−06	0.4248	5.70E−06	0.4079
cg00999243	chr19	808,526	*PTBP1*	Body	− 0.0134	5.99E−06	0.4248	5.61E−06	0.4079

*Chr*: Chromosome

*Gene*: CpGs are located in the gene if no distance is indicated (genome build Hg19)

**Feature**: Based on manifest feature annotation illumina

*Delta β coefficients*: computed from methylation *β*-values. *Negative β*-values indicate lower DNA methylation (hypomethylation) in cases compared with controls

*P*-values and FDR: Corresponding to M-values

#### Differentially methylated regions (DMRs) associated with the comprehensive lifestyle index

**Fig. 4:**
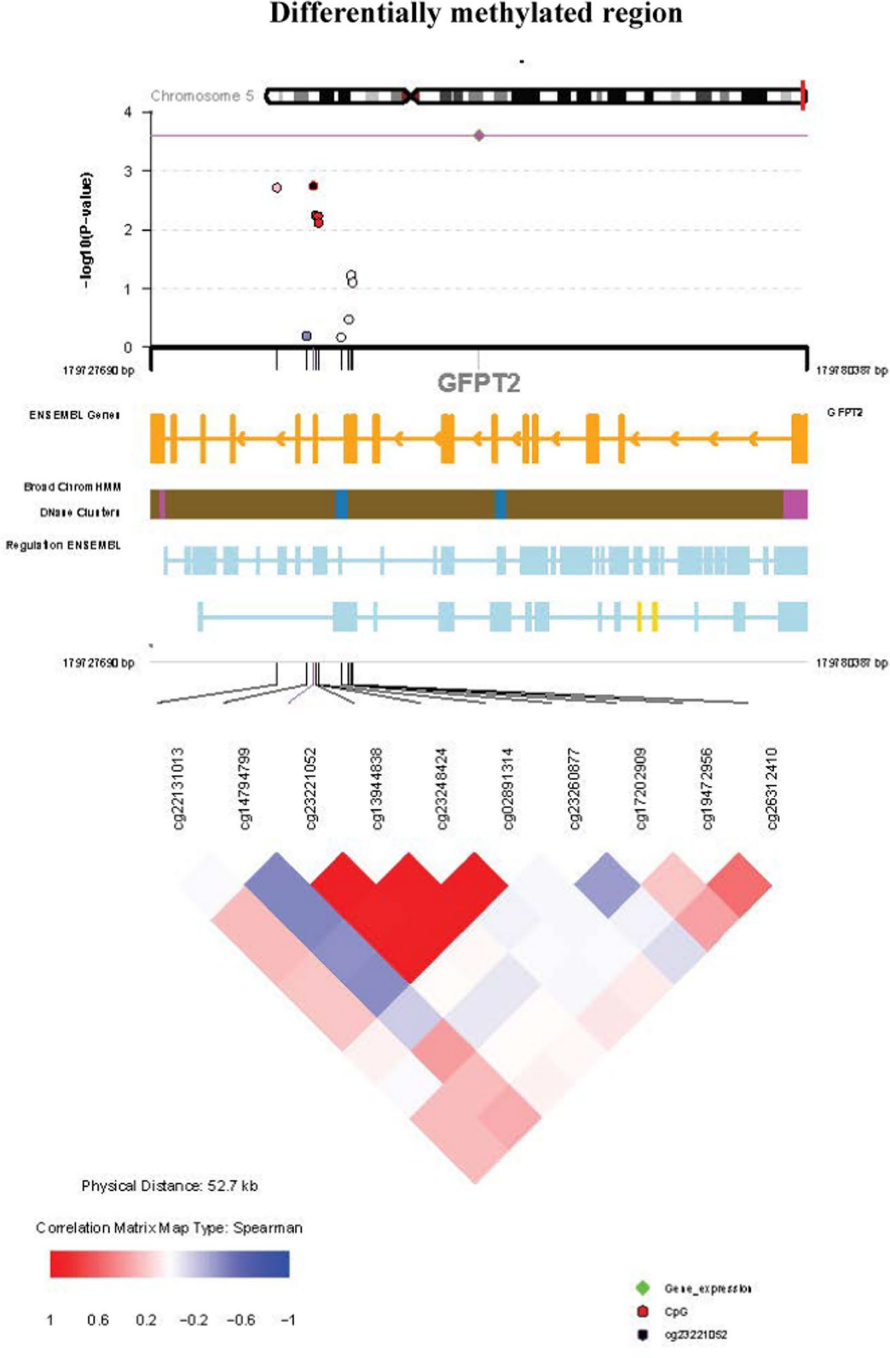
Regional plot (*Comet*) of the 52.7 kb region around the GFPT2 DMR. The plot includes three components: (1) The upper plot shows the strength and extent of the EWAS association signal, highlighting the EWAS phenotype association *p* values on a − log10 scale according to chromosomal position. (2) The middle panel provides customized annotation tracks; (3) the lower panel shows the comethylation between selected CpG sites in the genomic region

One genome-wide significant DMR was identified and annotated to the *GFPT2* gene (familywise error rate (FWER) = 0.036) on chromosome 5 (Fig. 4). The gene was hypermethylated, with 0.075 indicating the average difference in methylation in the bump. There were 10 CpG sites in this DMR, namely, cg26312410, cg19472956, cg17202909, cg23260877, cg02891314, cg23248424, cg13944838, cg23221052, cg14794799 and cg22131013. However, none of the CpGs overlapped with the top six DMPs.

#### Associations of DMPs and DMRs with T2DM and markers of glucose metabolism

None of the top DMPs from either the base or BMI-adjusted EWAS model were significantly associated with T2DM (OR < 1 and *p* values < 0.05). All the DMPs had an inverse association with T2DM, except for the *AHRR* DMP, which had a positive association with T2DM (Table 4). The associations between the DMPs and T2DM biomarkers showed that for every 1% increase in the methylation beta of the *ADCY7* DMP, the FBG was 0.29 mmol/L greater. The *LOXL2* gene had a similar effect size, but the direction of the effect was the opposite, with 0.28 mmol/L lower FBG for every 1% increase in methylation (Table 5). Finally, for every 1% increase in the methylation of the *LOXL2* and *PTBP1* DMPs, the HbA1c levels were 1.8 mmol/mol and 2.9 mmol/mol lower, respectively (Table 6). There was no significant association between the identified DMR and T2DM or between the identified DMR and biomarkers of T2DM.

**Table 4:** Logistic regression results of the top 6 differentially methylated positions, comprehensive lifestyle index and lifestyle factor scores for patients with T2DM The beta values used were inversely normally transformed

	Base model			Adjusted model		
	OR	95%CI	*P* value	OR	95%CI	*P* value
cg23604683	0.98	0.84, 1.13	0.8	0.98	0.84, 1.14	0.8
cg02879453	0.97	0.84, 1.13	0.7	0.99	0.84, 1.16	0.9
cg21774457	0.99	0.85, 1.15	0.9	0.98	0.84, 1.14	0.8
cg26703534	1.03	0.88, 1.19	0.7	1.02	0.88, 1.20	0.8
cg04028450	0.92	0.79, 1.07	0.3	0.92	0.79, 1.07	0.3
cg00999243	0.88	0.76, 1.02	0.10	0.88	0.76, 1.03	0.10
CLI	1.29	0.92, 1.81	0.15	1.25	0.88, 1.78	0.2
Smoking	1.11	0.76, 1.60	0.6	1.12	0.77, 1.63	0.5
Alcohol intake	1.08	0.92, 1.26	0.3	1.08	0.92, 1.27	0.3
Physical activity	1.23	1.03, 1.46	**0.020**	1.24	1.04, 1.47	**0.015**
DQII	0.78	0.35, 1.74	0.5	0.77	0.35, 1.73	0.5

Numbers in bold indicate statistically significant values (*P* ≤ 0.05)

*OR:* odds ratio

*CI:* confidence interval

*CLI:* comprehensive lifestyle index

*DQII:* Diet Quality Index International

**Table 5:** Linear regression results of the top 6 differentially methylated positions, comprehensive lifestyle index and lifestyle factor scores for FBG as an outcome

	Base model			Adjusted model		
	Beta	95%CI	*P* value	Beta	95%CI	*P* Value
cg23604683	− 0.01	− 0.26, 0.24	> 0.9	− 0.04	− 0.28, 0.21	0.8
cg02879453	0.29	0.04, 0.54	**0.024**	0.20	− 0.06, 0.47	0.13
cg21774457	− 0.08	− 0.33, 0.17	0.6	− 0.03	− 0.28, 0.22	0.8
cg26703534	0.09	− 0.16, 0.34	0.5	0.08	− 0.17, 0.33	0.5
cg04028450	− 0.28	− 0.53, − 0.04	**0.023**	− 2.8	− 0.52, − 0.03	**0.025**
cg00999243	− 0.18	− 0.43, 0.08	0.2	− 0.15	0.40, 0.10	0.2
CLI	0.22	− 0.35, 0.79	0.5	0.14	− 0.44, 0.72	0.6
Smoking	0.10	− 0.5, 0.71	0.8	0.12	− 0.49, 0.74	0.7
Alcohol intake	0.06	− 0.20, 0.32	0.7	0.07	− 0.20, 0.33	0.6
Physical activity	0.30	0.02, 0.59	**0.036**	0.32	0.03, 0.60	**0.031**
DQII	− 0.34	− 1.7, 1.0	0.6	− 0.36	− 1.7, 1.0	0.6

Numbers in bold indicate statistically significant values (*P* ≤ 0.05)

The beta values used were inversely normally transformed

*CI:* confidence interval

Beta:  Estimated coefficient, i.e., degree of change in the outcome variable for every unit change in the predictor variable

*CLI:* comprehensive lifestyle index

*DQII:* Diet Quality Index International

*FBG:* fasting blood glucose in mmol/L

**Table 6:** Linear regression results of the top 6 differentially methylated positions, comprehensive lifestyle index and lifestyle factor scores for HbA1c

	Base model			Adjusted model		
	Beta	95%CI	*P* value	Beta	95%CI	*P* value
cg23604683	− 0.89	− 2.7, 0.90	0.3	− 1.0	− 2.8, 0.76	0.3
cg02879453	0.59	− 1.2, 2.4	0.5	0.45	− 1.5, 2.4	0.6
cg21774457	− 1.2	− 3.0, 0.59	0.2	− 1.0	− 2.8, 0.75	0.3
cg26703534	− 0.36	− 2.1, 1.4	0.7	− 0.31	− 2.1, 1.5	0.7
cg04028450	− 1.7	− 3.5, 0.03	0.054	− 1.8	− 3.5, − 0.01	**0.049**
cg00999243	− 2.9	− 4.7, − 1.1	**0.002**	− 2.8	− 4.6, − 1.0	**0.003**
CLI	0.65	− 3.5, 4.8	0.8	0.38	− 3.8, 4.6	0.9
Smoking	− 0.05	− 4.5, 4.4	> 0.9	− 0.05	− 4.5, 4.4	> 0.9
Alcohol intake	0.51	− 1.4, 2.4	0.6	0.55	− 1.4, 2.5	0.6
Physical activity	0.83	− 1.2, 2.9	0.4	0.86	− 1.2, 2.9	0.4
DQII	− 0.23	− 10, 9.7	> 0.9	− 0.25	− 10, 9.7	> 0.9

Numbers in bold indicate statistically significant values (*P* ≤ 0.05)

The beta values used were inversely normally transformed

*CI:* confidence interval

*Beta:* Estimated coefficient, i.e., degree of change in the outcome variable for every unit change in the predictor variable

*CLI:* comprehensive lifestyle index

*DQII:* Diet Quality Index International

#### Enrichment analyses and annotation

The results from 5 enrichment sections were obtained from the EWAS toolkit [46]. These included trait enrichment, gene ontology enrichment (GO), motif enrichment, genomic location and Kyoto Encyclopedia of Genes and Genomes (KEGG) enrichment. Four annotation sections were also retrieved, including methylation and expression regulation annotation, histone modification and chromatin state. In brief, smoking was the most significantly enriched trait among the top 100 CpG sites (Supplementary Fig. 4). DNA methylation sites related to the lifestyle index were highly enriched in the 3’UTR regions and on the islands (Supplementary Fig. 5).

There was tissue-specific annotation of the CpG sites and significant variation in the DNA methylation levels of the top six sites in different tissues (Supplementary Table 1). The relationship between the methylation level of the CpG sites and the expression of nearby genes was also clear (Supplementary Table 2 and Fig. 6a–k). A network visualization for exploring and understanding the hierarchical associations between genes and traits highlighted smoking, smoking cessation, blood HbA1c levels, fruit consumption and obesity as key traits closely interlinked with T2DM (Supplementary Fig. 7).

#### Mediation analyses

**Fig. 5:**
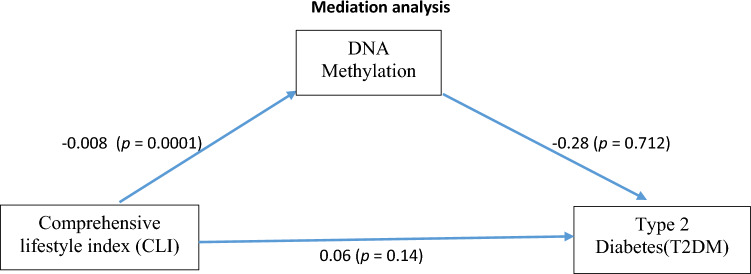
The effect of CLI on T2DM status was not fully mediated via DNA methylationDNA methylation. As the figure illustrates, the direct effect of CLI on DNA methylation was significant (*p* value = 0.0001). However, the regression coefficient between the CLI and T2DM and the regression coefficient between DNA methylation and T2DM was not significant. The indirect effect was (− 0.008) (− 0.28) = 0.0022, and the significance of this indirect effect was tested by bootstrapping 1000 times at a 95% confidence interval (− 0.01 to 0.02). Thus, the indirect effect was not statistically significant (*p* value = 0.72)

The effect of the lifestyle index on T2DM status was not fully mediated via DNA methylation. The direct effect of the lifestyle index on DNA methylation was significant (*p* value = 0.0001). For every unit increase in the lifestyle index, there was a 0.0008% decrease in the methylation beta of the top six DMPs. However, every unit increase in methylation of the top 6 DMPs led to a 0.28% decrease in the likelihood of developing T2DM. This difference was, however, not significant. As illustrated in Fig. 5, the indirect effect was (− 0.008)*(− 0.28) = 0.0022. The significance of these indirect effects was tested by bootstrapping 1000 times at a 95% confidence interval (− 0.01 to 0.02). Thus, the indirect effect was not statistically significant (*p* value = 0.72).

## Discussion

This explorative epigenome-wide association study is the first to report the associations of multiple lifestyle factors and a comprehensive lifestyle index combined with DNA methylation, T2DM and markers of T2DM in a SSA population. Herein, we highlight three key findings: 1)We identified one novel candidate locus (DMP); and 2) one region (DMR), associated with a lifestyle index not previously reported with individual lifestyle factors for T2DM; and 3), we identified associations between three DMPs and markers of T2DM (FBG and HbA1c).

Our study adds to the limited number of studies evaluating the effects of multiple lifestyle factors in combination. A lifestyle index derived from multiple lifestyle factors is better suited for differentiating individuals with healthy versus unhealthy lifestyles compared to individual lifestyle factors [13]. Lifestyle factors can alter DNA methylation, and an overall effect of lifestyle factors on DNA methylation can easily be achieved through a comprehensive lifestyle index instead of focusing on single factors. This has also been demonstrated by Klemp et al. who created a lifestyle score and used it as a discovery tool for DNA methylation patterns that drive obesity in a European population [13].

In the present study, based on the constructed lifestyle index, we identified one genome-wide significant DMP, cg23604683, annotated to an intergenic region. However, there is limited information about this intergenic region. Its role in diabetes etiology and its significant interaction with modifiable risk factors, except for BMI, have not been fully explored. Sharp et al. investigated maternal BMI at the start of pregnancy. They identified this genome-wide significant DMP as one of those differentially methylated by means of epigenome-wide DNA methylation analyses in whole blood from offspring, reporting an association with a beta value of − 0.00062, an SE of 1e − 04 and a p value of 5.1 × 10^−10^ [48]. Although in an infant population, this finding supports our findings since BMI is strongly influenced by dietary patterns and physical activity, which are part of our lifestyle index [49–51].

Although not genome-wide significant, several of the DMPs with the lowest nominal pvalues for their association with the lifestyle index have previously been associated with individual lifestyle factors in other studies. For instance, the *ADCY7* gene (cg02879453) has been implicated as an important component of the molecular pathways leading to alcohol use and dependence [52]. Moreover, its knockout is associated with stimulation of the insulin secretion pathway and subsequent hyperinsulinemic hypoglycaemia [53]. This may explain the site’s significant positive association with blood glucose levels in the present study.

In a study in which continuous alcohol usage was considered a predictor variable for DNA methylation in a population including individuals of African ancestry, the *SMARCE1* gene (cg21774457) was significant (beta value =  − 0.000245, *p* value = 9.4 × 10^−5^) [54]. In addition, this gene has been implicated in the comparison of smoking-related DNA methylation levels between adults and newborns from prenatal exposure in a meta-analysis (beta value =  − 0.00364, *p* value = 7.5 × 10^−5^) [55]. Although small, the beta values are likely to have a functional impact on disease pathways, especially if the gene is enriched for localization to island shores, DNase I hypersensitivity sites, enhancers and TF binding sites, since methylation at these sites is quite dynamic [56]. Methylation of the *SMARCE1* gene (cg21774457) could thus be a biomarker for smoking and alcohol intake. In addition, the gene has been associated with the type 1 diabetes mellitus phenotype [57–59], suggesting that it may impact insulin secretion regulation.

The methylation of sites annotated to the *AHRR* gene (cg26703534) has been consistently and specifically associated with smoking-related traits in several epigenome-wide association studies, including studies of adult smoking and maternal smoking during pregnancy, in diverse populations [60–68]. It is thus a well-known smoking epigenetic signature. Its association (FDR = 0.00010) with smoking status was also noted in the same meta-analysis that reported the *SMARCE1* gene (cg21774457) [55]. Additionally, similar to the DMP annotated to the *SMARCE1* gene (cg21774457), a DMP (cg26703534) annotated to the AHRR gene was also differentially methylated (*p* value =  < 1 × 10^−4^) and associated with continuous alcohol usage in the same meta-analysis of pooled samples, including those of African ancestry [54]. The association between DMP annotated to the AHRR gene (cg26703534) and lifestyle index in our Ghanaian study population was therefore likely driven by smoking.

However, differential methylation of this gene may not be exclusively driven by smoking. Differential methylation of the 3’ UTR has additionally been associated with body fat distribution [69], BMI-adjusted waist circumference [70, 71] and BMI-adjusted hip circumference [71, 71] phenotypes. These phenotypes are directly influenced by dietary patterns and physical activity levels in the lifestyle index. A recent study examining DNA methylation in healthy versus unhealthy individuals also identified differentially methylated sites in this gene [13]. In the present study, the *AHRR* gene (cg26703534) showed a nonsignificant association with T2DM, blood glucose and HbA1c levels. However, differential methylation of the gene body at the S shelf island is significantly associated with gestational diabetes mellitus and type 1 diabetes disorders [72, 73].

The *LOXL2* gene (cg04028450) was previously reported to be significantly hypomethylated with BMI as an exposure factor [74–76]. In addition, this observation has also been made with smoking as an exposure [55, 77]. Thus, smoking status and BMI, which are included in the lifestyle index constructed in this study, drive changes in the DNA methylation of the *LOXL2* gene (cg04028450). Although there was no significant association between *LOXL2* (cg04028450) and T2DM, it was significantly associated with blood glucose levels and HbA1c according to the adjusted model. Nonetheless, according to Dongiovanni and colleagues, *LOXL2* (cg04028450) is associated with T2DM, and its upregulation has been observed in nonalcoholic fatty liver disease patients with T2DM [78]. Furthermore, *LOXL2* (cg04028450) is associated with hyperglycemia [79], giving weight to the present study’s observed significant association with blood glucose levels and HbA1c.

In a recent study investigating the potential of the *PTBP1* gene (cg00999243) to predict intracranial aneurysms, findings showed that long-term exposure to tobacco smoke leads to hypermethylation of its promoter region [80]. It is, therefore, a potential predictive marker of smoking, a component of the lifestyle index. In the present study, this gene showed no significant association with T2DM but was significantly associated with HbA1c. Previously, it was reported that in nondiabetic subjects, SNPs in introns of the *PTBP1* gene (cg00999243), including rs123698 and rs736926, were associated with proinsulin conversion and insulin secretion [81]. Additionally, *PTBP1* (cg00999243) is an important gene involved in beta-cell function because it influences glucose-stimulated insulin secretion [82, 83]. Therefore, this gene is likely a significant marker of hyperglycemia, and its significant association with HbA1c in the present study supports these previously reported findings.

The identified genome wide significant DMR annotated to the *GFPT2* gene was highly hypermethylated but showed no significant association with T2DM, HbA1c, or blood glucose levels. However, past studies have implicated this gene in diabetes and all type 2 diabetes mellitus phenotypes [84]. One such recent study reported hypermethylation and significant correlation of the expression of the GFPT2 gene with enrichment in the insulin resistance and insulin signalling pathways, suggesting that this gene is a suitable candidate biomarker for metabolic syndrome [85]. In addition, a study on DNA methylation patterns reflecting the lifestyles of individuals reported a DMR annotated to this gene to be hypomethylated in individuals who were healthy versus those with unhealthy lifestyles [13]. The involvement of this DMR in lifestyle factors is thus consistent with findings from other studies. Nevertheless, the absence of a significant association with T2DM, HbA1c, or blood glucose levels could be attributed to the limited sample size and ethnic variations.

## Strengths and limitations

The primary strength of this study lies in the creation of a lifestyle index that integrates various lifestyle factors and examines their association with DNA methylation in SSA, a population often underrepresented in epigenetics research.. This study is not without limitations. Although this is the largest lifestyle EWAS in SSA populations to date, the sample size provided limited statistical power. Furthermore, the large environmental and genetic heterogeneity among SSA populations makes it difficult to generalize these findings. However, the selected study population is unique, and the present study is the first EWAS study focusing on the associations among multiple lifestyle factors in SSA. Nonetheless, further investigations with larger sample sizes in diverse SSA populations are warranted. For lifestyle factors, our scoring system was based on self-report questionnaires, for which we cannot exclude response bias. This was countered by in-depth research and the inclusion of previous studies that focused on individual lifestyle factors as exposures to ensure consistency. Most of these studies also implicated the majority of the identified DMPs.

## Conclusions

The novel DNA methylation locus (cg23604683) and region (*GFPT2)* identified in this study suggest that lifestyle factors might have a combined effect on DNA methylation, adding to the growing body of literature aimed at understanding the determinants and underlying mechanisms of susceptibility of SSA populations to T2DM.

## Supplementary Information

Additional file 1.

## Data Availability

The RODAM dataset analysed for the current study is available upon reasonable request from the PI of the RODAM study (c.o.agyemang@amsterdamumc.nl)
