# Supplementary material for: A comprehensive lifestyle index and its associations with DNA methylation and type 2 diabetes among Ghanaian adults: the rodam study
Source: Clin Epigenetics. 2024 Oct 16;16:143. doi: 10.1186/s13148-024-01758-z (PMC11481717; doi:10.1186/s13148-024-01758-z)
Supplement: Supplementary file 1 — Additional file 1. [file 13148_2024_1758_MOESM1_ESM.docx]

**Supplementary materials**

**Figure 1:** Flow chart of sample selection. The cases and controls were those with and without T2DM.

Agreed to participate (n = 6,385)

## Recruitment

Included (n = 5,898)

Excluded (n = 487)

- Missing physical examination (n = 456)
- Missing blood sample (n = 21)

## Analysis

Missing or implausible values for:

- Ghana-Food Propensity Questionnaire (n = 1,238)
- Age >70 years or <25 years (n = 105)
- Body Mass Index (n = 7)
- Waist circumference (n = 2)
- Physical activity (MET-h/wk) (n = 305)
- Smoking status (n = 56)
- Educational status (n = 12)
- Total energy intake ≥ 95^th^ percentile: 4,934 kcal/d) (n = 181)

Missing or implausible values for:

- Ghana-Food Propensity Questionnaire (n = 117)
- Age >70 years or <25 years (n = 9)
- Body Mass Index (n = 1)
- Waist circumference (n = 1)
- Physical activity (MET-h/wk) (n = 31)
- Smoking status (n = 6)
- Educational status (n = 1)
- Total energy intake ≥ 95^th^ percentile: 4,934 kcal/d) (n = 16)

Analysed (n = 3,451)

Analysed (n = 359)

Ineligible because their DNA was not profiled (n=5,162)

Epigenetics Subset (n=736)

Controls (n=471)

Cases (n= 265)

Methylation profiling, processing, and quality control

Methylation profiling, processing, and quality control

Controls (n=426)

Cases (n= 287)

Missing data:

Smoking = 21

Physical activity = 76

Waist measurement = 2

Energy = 123

Components of DQII = 184

Multiple Imputation (n=10)

Cases (n=287)

Controls (n=426)

**(a) (b)**


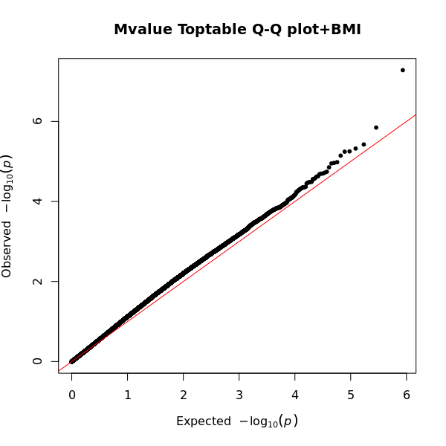

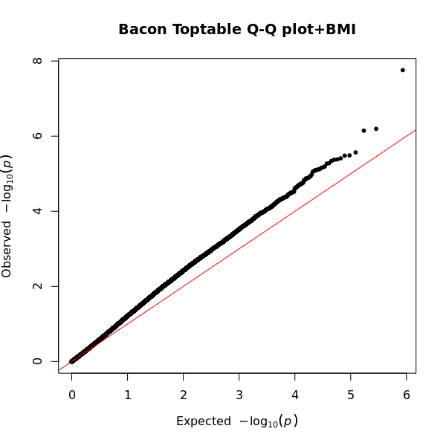


**(c) (d)**


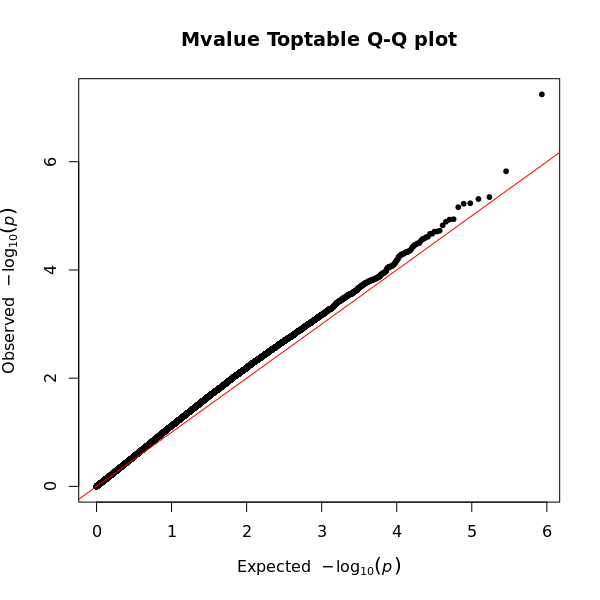

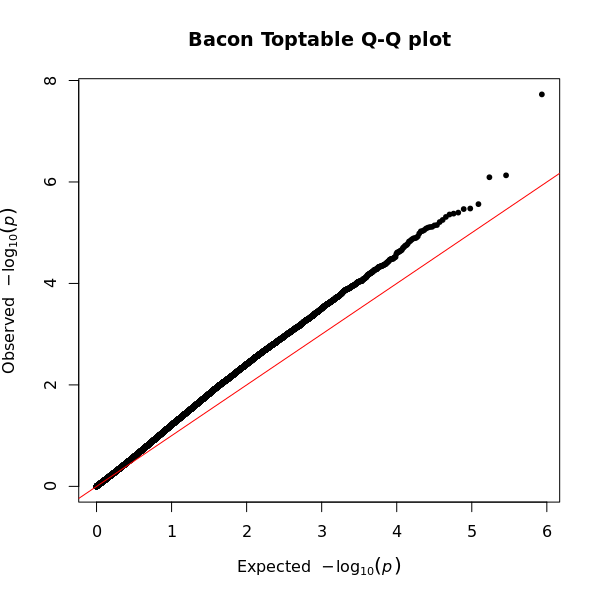


**Figure 2:** a) Q‒Q plot of EWAS p values for DMP analyses of T2DM patients adjusted for BMI after bacon correction. b) Q‒Q plot of EWAS p values for DMP analyses of T2DM patients adjusted for BMI for Mvalues. c) Q‒Q plot of EWAS p values for DMP analyses of type 2 diabetes patients unadjusted for BMI after bacon correction. d) Q‒Q plot of EWAS p values for DMP analyses of T2DM patients adjusted for BMI for Mvalues. The x axes represent the theoretical quantiles, while the y axes represent the observed values. The red line represents a normal distribution.

1. **Model 1a. b) Model 1b.**


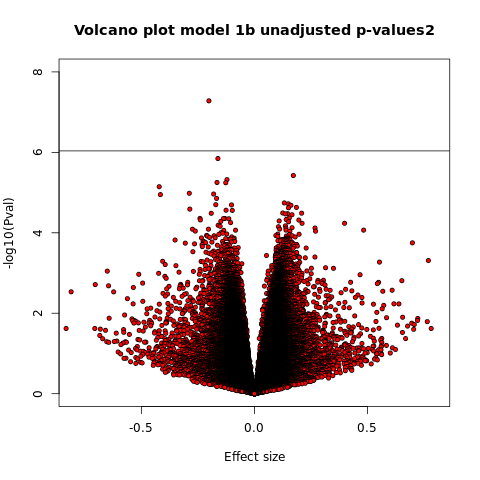

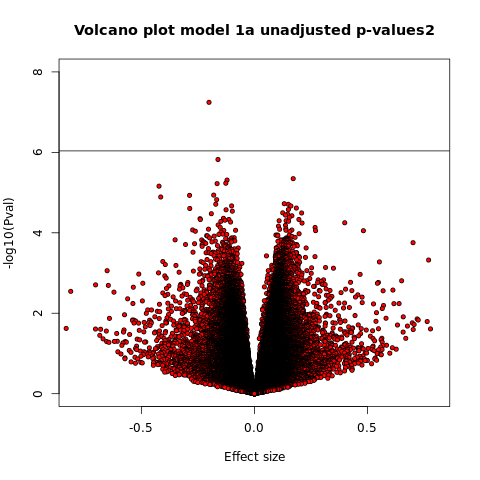


**Figure 3:** Volcano plots showing the effect sizes of the p values. Model 1a: Unadjusted p values without BMI; Model 1b: Unadjusted p values with BMI. The plots enabled the identification of genes with large fold changes that were also statistically significant. These may be the most biologically significant genes. The most upregulated genes are on the right, the most downregulated genes are on the left, and the most statistically significant genes are on the top. The horizontal line represents the genome-wide significance level (FDR < 0.05).

**Enrichment analysis of the top 100 DMPs**

1. **Trait enrichment**


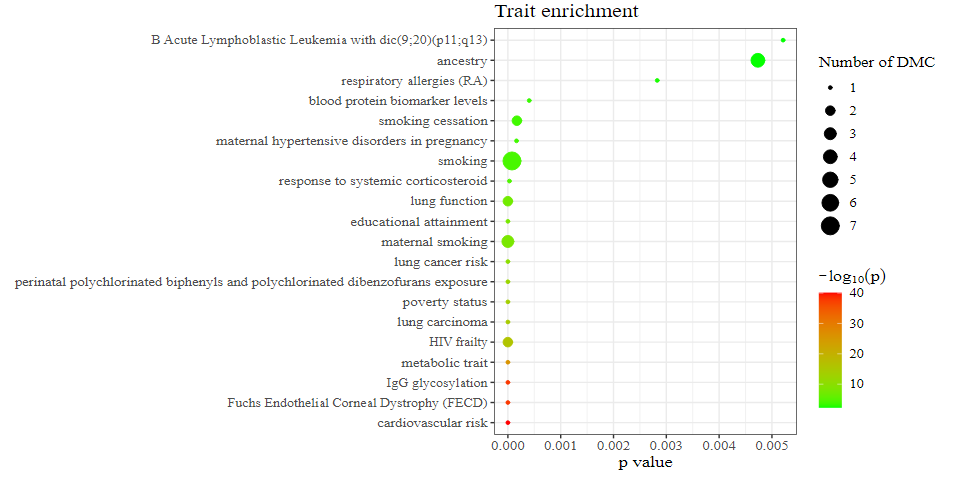


**Figure 4:** Specific traits that were significantly enriched. The most significantly enriched trait was smoking

1. **Genomic location enrichment**


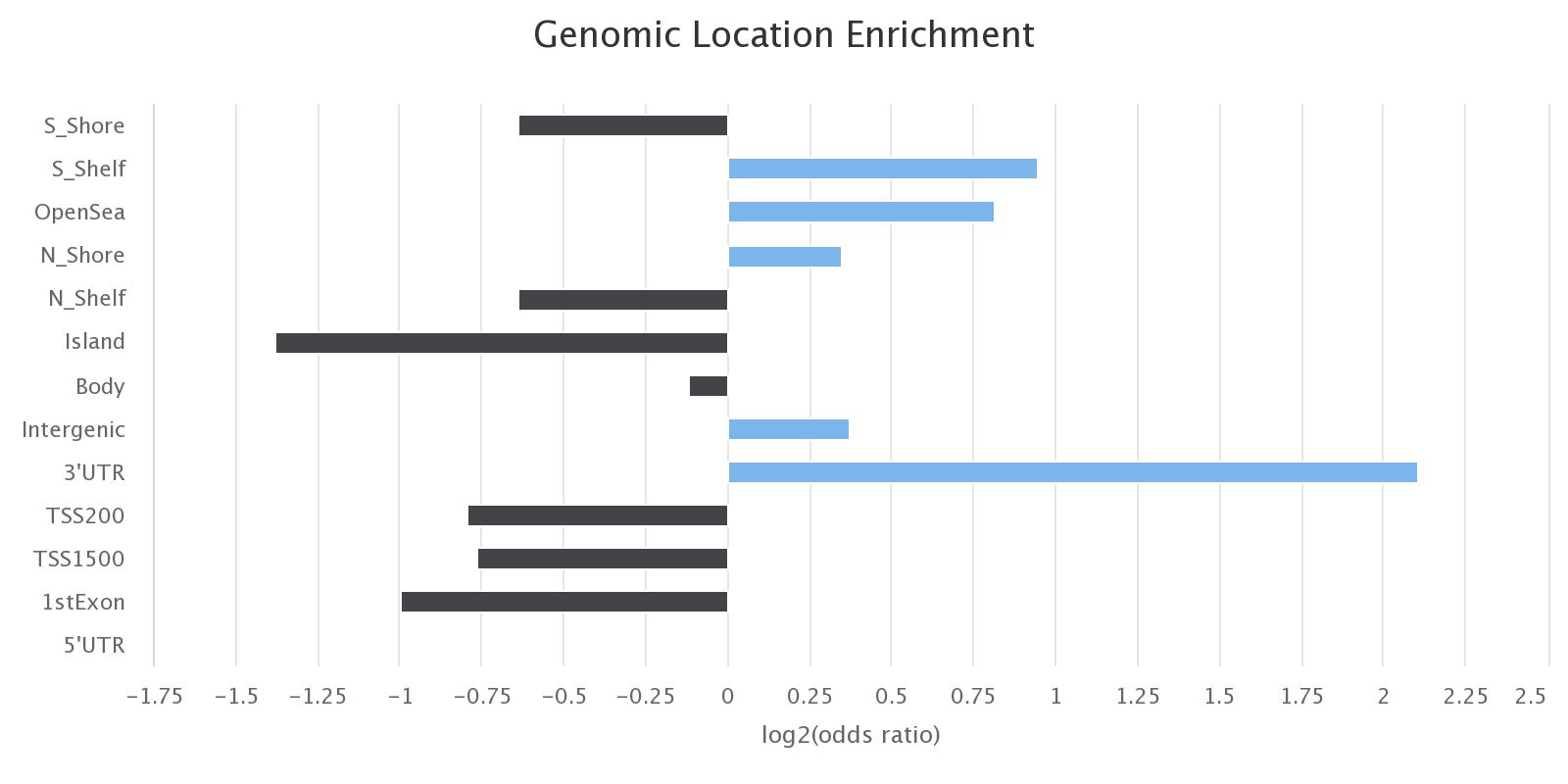


**Figure 5:** DNA methylation sites related to T2DM are highly enriched in 3'UTR regions and on islands.

**Tissue Methylation**

**Table 1:** Methylation levels across tissues of the top 6 DMPs

| **Probe** | **tau (hyper)** | **tau (hypo)** | **tau (\|hyperhypo\|)** | **Tissue with lowest methylation** | **Tissue with highest methylation** |
| --- | --- | --- | --- | --- | --- |
| **cg23604683** | 0.301 | 0.518 | 0.217 | brain-cerebellum | bone marrow |
| **cg02879453** | 0.083 | 0.581 | 0.497 | brain-cerebellum | fallopian tube |
| **cg21774457** | **N/A** | **N/A** | **N/A** | **N/A** | **N/A** |
| **cg26703534** | 0.061 | 0.642 | 0.58 | blood | brain-cerebrum |
| **cg00999243** | 0.081 | 0.646 | 0.564 | pituitary | bone marrow |
| **cg04028450** | 0.375 | 0.544 | 0.169 | brain-cerebellum | blood |

**Table 2:** DNA methylation and expression levels of the top 6 DMPs according to the EWAS Atlas indicating changes in expression levels in different tissues. The results included correlations with the expression of the genes in other tissues.

| **Probe** | **Gene** | **Gene Symbol** | **Location** | **Pearson Correlation** | **P - Value** | **Tissue** | **Data source** |
| --- | --- | --- | --- | --- | --- | --- | --- |
| **cg23604683** | **N/A** | **N/A** | **N/A** | **N/A** | **N/A** | **N/A** | **N/A** |
| cg02879453 | ENSG00000121281 | ADCY7 | body | -0.227 | 4.54e-9 | kidney | TCGA-KIRP,TCGA-KIRC,TCGA-KICH |
| **cg21774457** | **N/A** | **N/A** | **N/A** | **N/A** | **N/A** | **N/A** | **N/A** |
| cg26703534 | ENSG00000063438 | AHRR | body | 0.207 | 3.45e-5 | liver | TCGA-LIHC |
| cg26703534 | ENSG00000063438 | AHRR | body | 0.226 | 3.84e-5 | stomach | TCGA-STAD |
| **cg26703534** | ENSG00000063438 | AHRR | body | 0.55 | 4.88e-12 | testis | TCGA-TGCT |
| cg00999243 | ENSG00000011304 | PTBP1 | body | -0.146 | 8.16e-3 | stomach | TCGA-STAD |
| **cg00999243** | ENSG00000011304 | PTBP1 | body | -0.286 | 7.73e-4 | testis | TCGA-TGCT |
| cg04028450 | ENSG00000134013 | LOXL2 | body | -0.453 | 3.44e-8 | testis | TCGA-TGCT |
| cg04028450 | ENSG00000134013 | LOXL2 | body | -0.367 | 1.44e-19 | brain | TCGA-GBM,TCGA-LGG |
| cg04028450 | ENSG00000253837 | RP11-177H13.2 | body | -0.239 | 5.25e-3 | testis | TCGA-TGCT |
| cg04028450 | ENSG00000253837 | RP11-177H13.2 | body | -0.117 | 5.06e-3 | brain | TCGA-GBM,TCGA-LGG |
| cg04028450 | ENSG00000253837 | RP11-177H13.2 | body | -0.106 | 6.74e-3 | kidney | TCGA-KIRP,TCGA-KIRC,TCGA-KICH |

**a) CpG2 - cg02879453 b) CpG 4 - cg26703534**

**in Liver tissue**


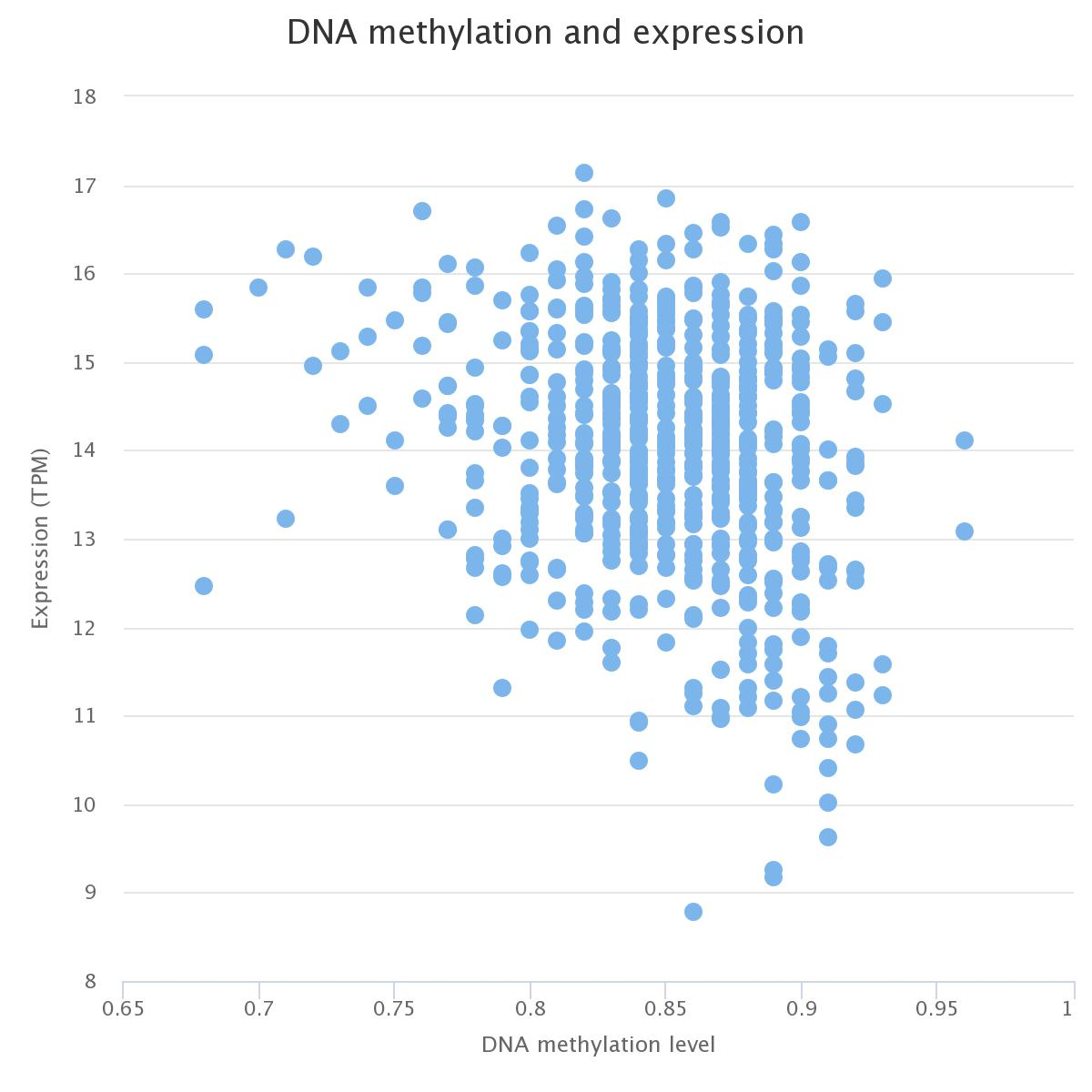

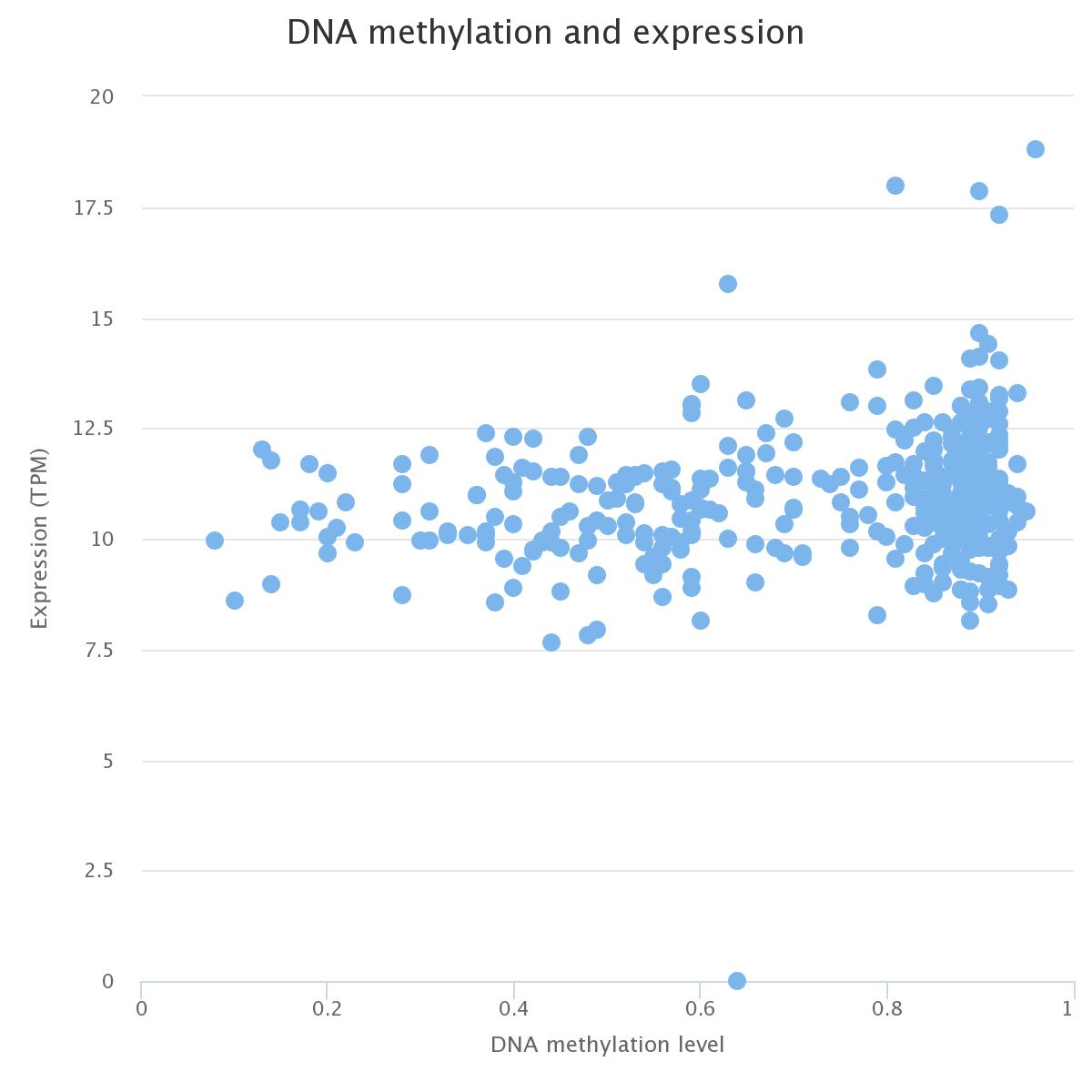


**c) CpG 4 - cg26703534 in d) CpG 4 - cg26703534**

**Stomach tissue in Testis tissue**


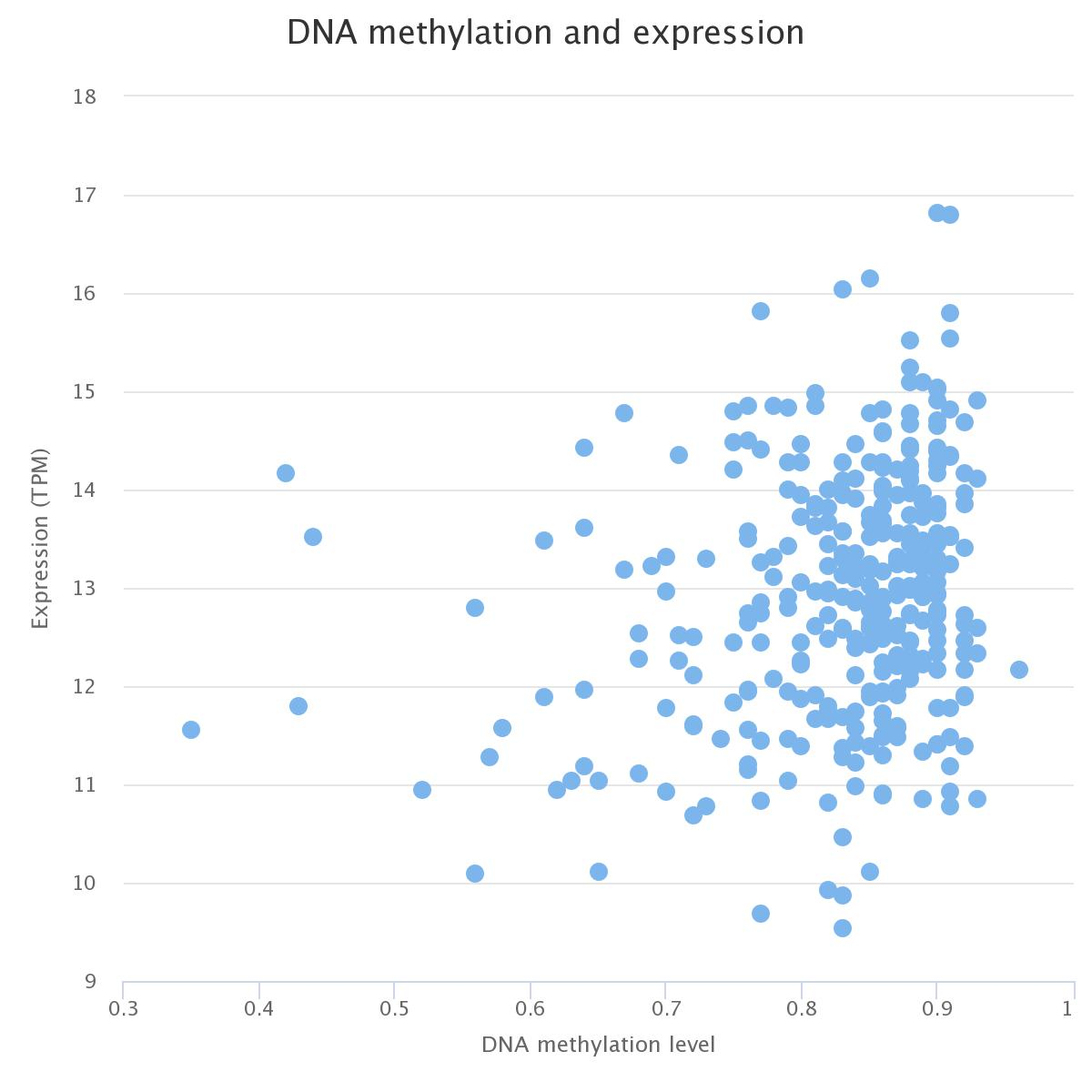

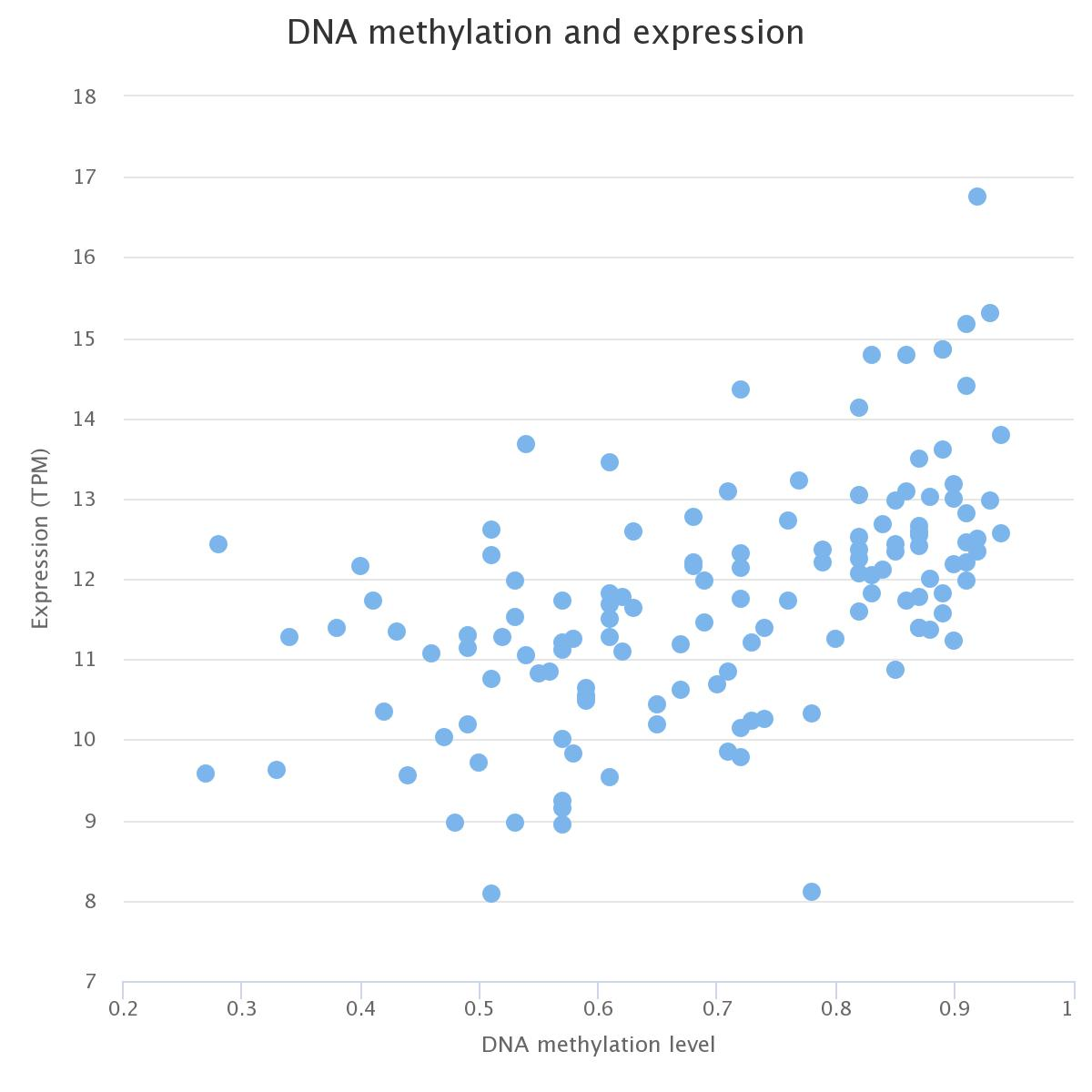


**e) CpG 5 - cg00999243 f) CpG 5 - cg00999243**

**In Stomach tissue In Testis tissue**


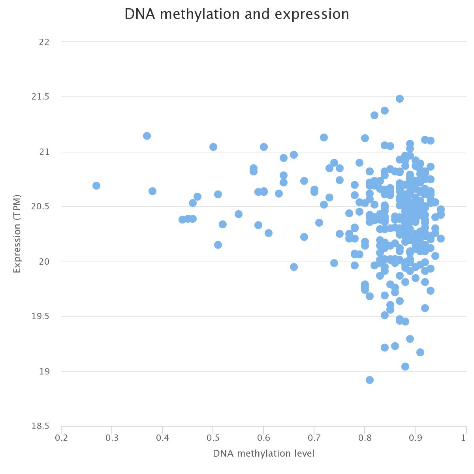


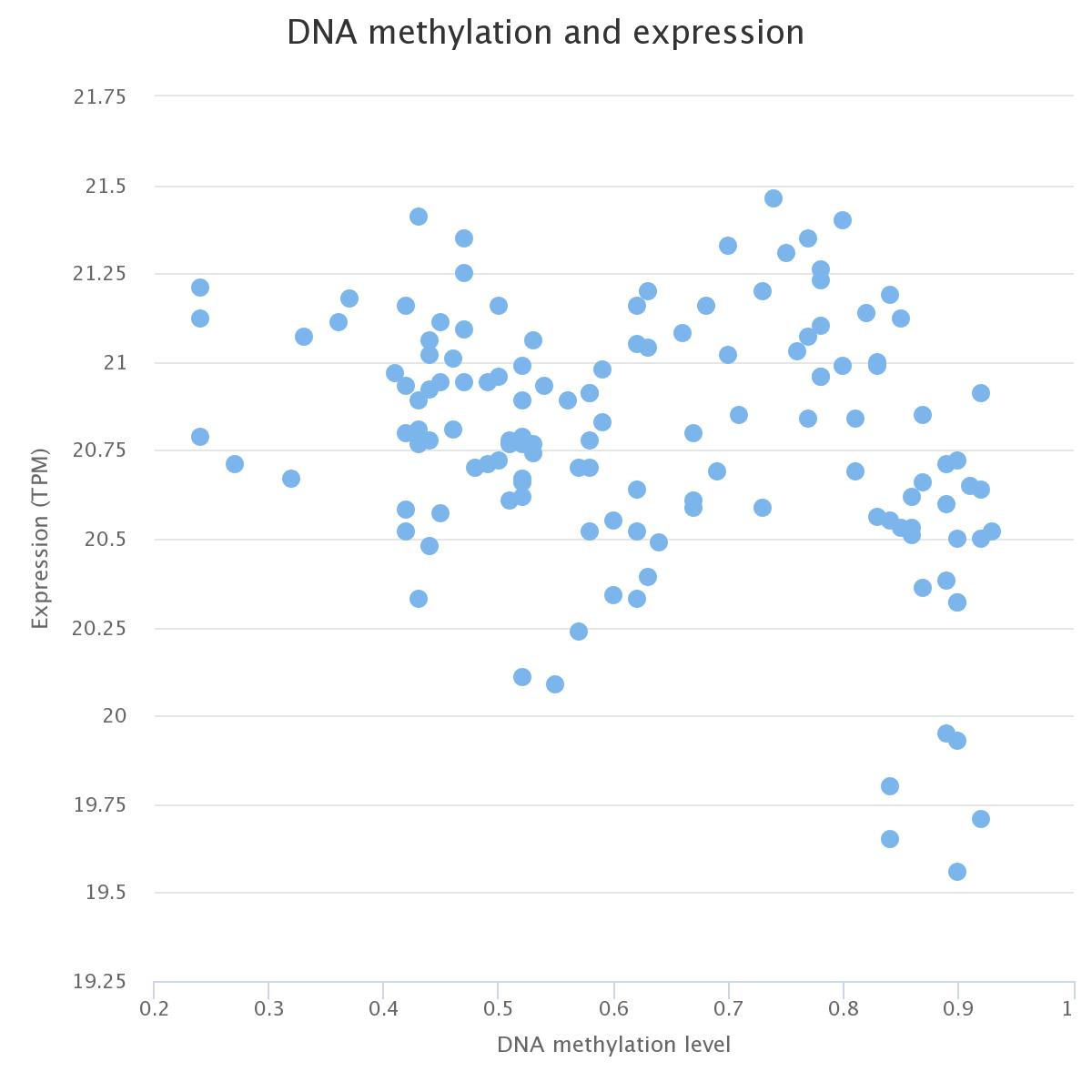


g) **CpG 6 - cg04028450**  h) **CpG 6 - cg04028450**

**In Testis In Testis – different gene symbol**


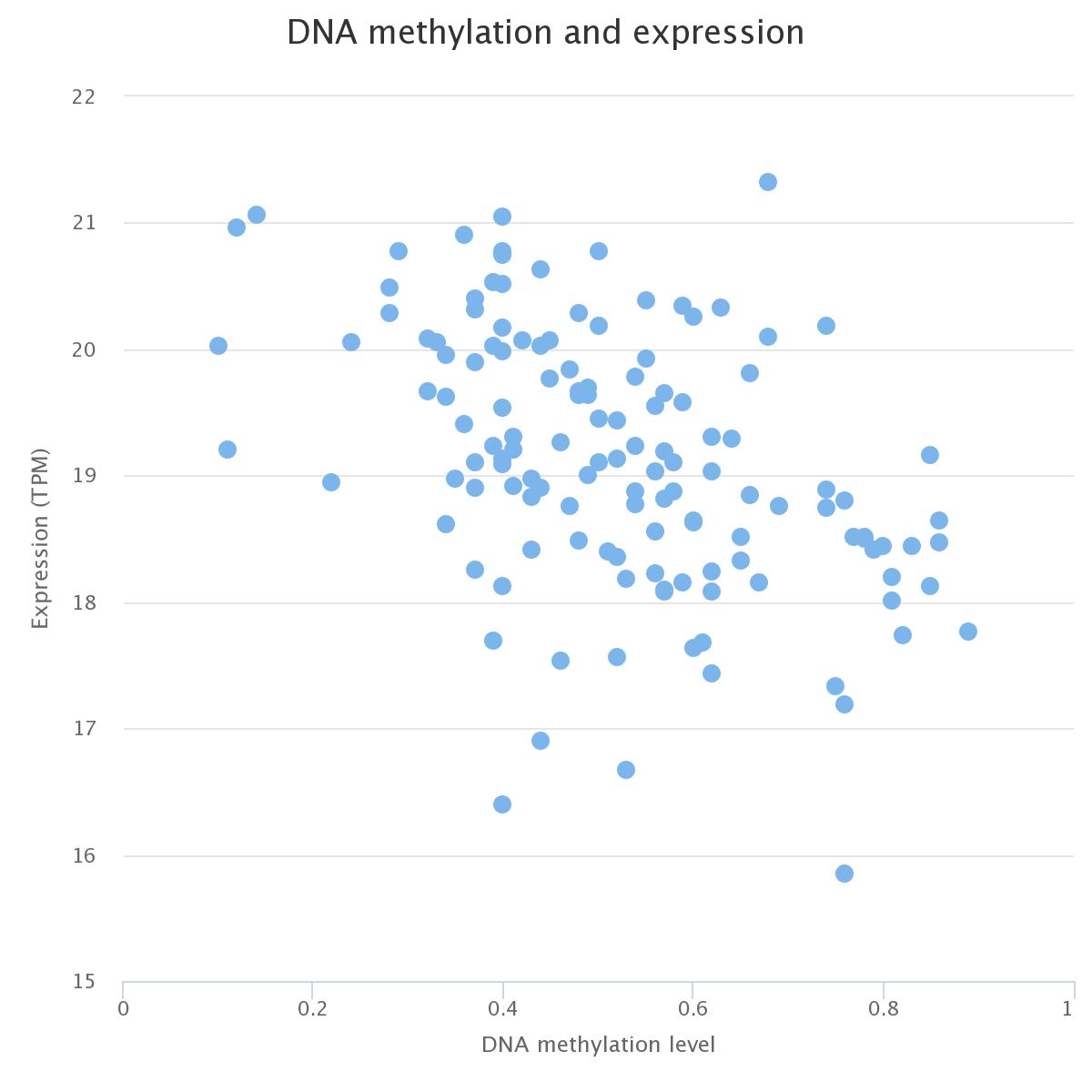

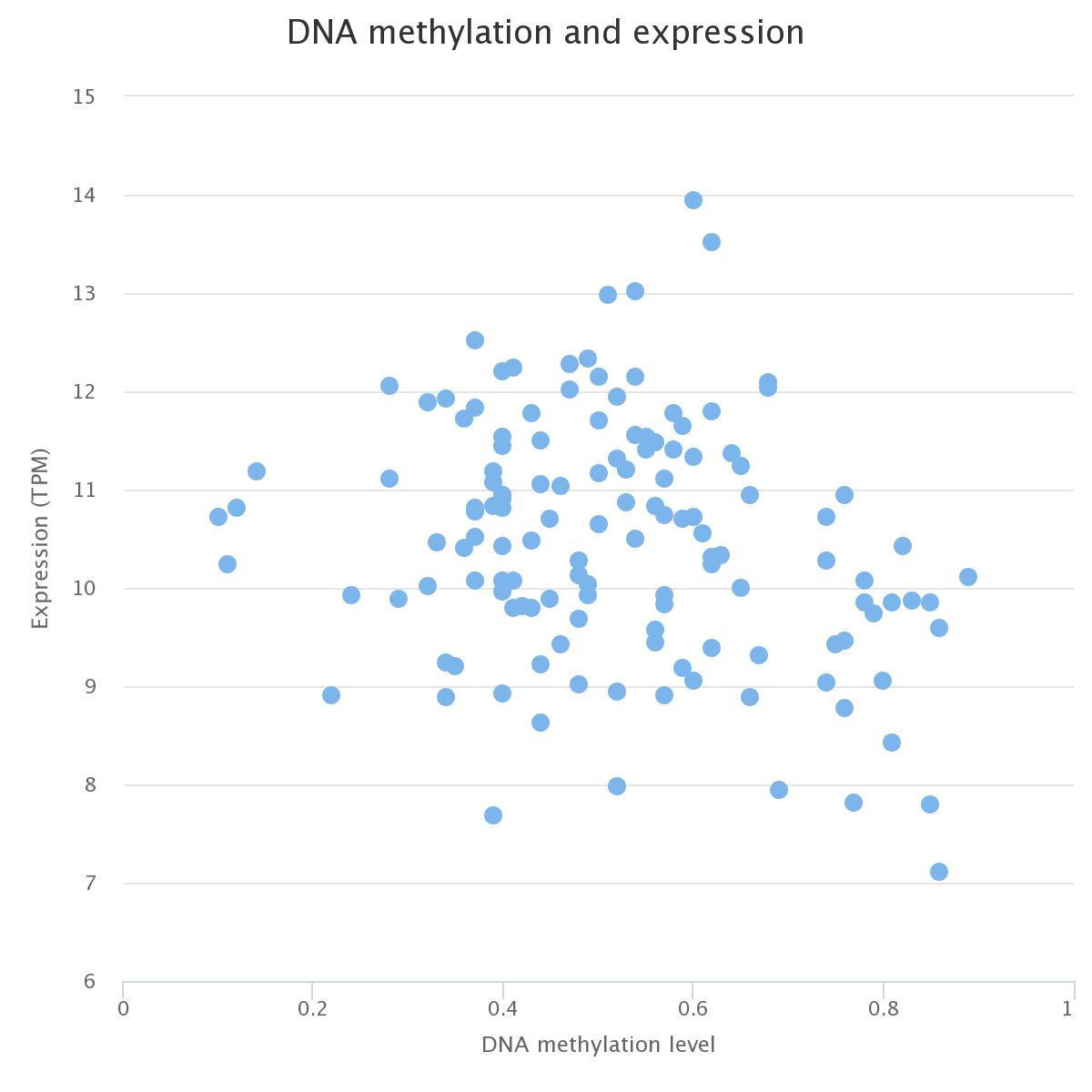


i) **CpG 6 - cg04028450**  j) **CpG 6 - cg04028450**

**In Brain In Brain – different gene symbol**


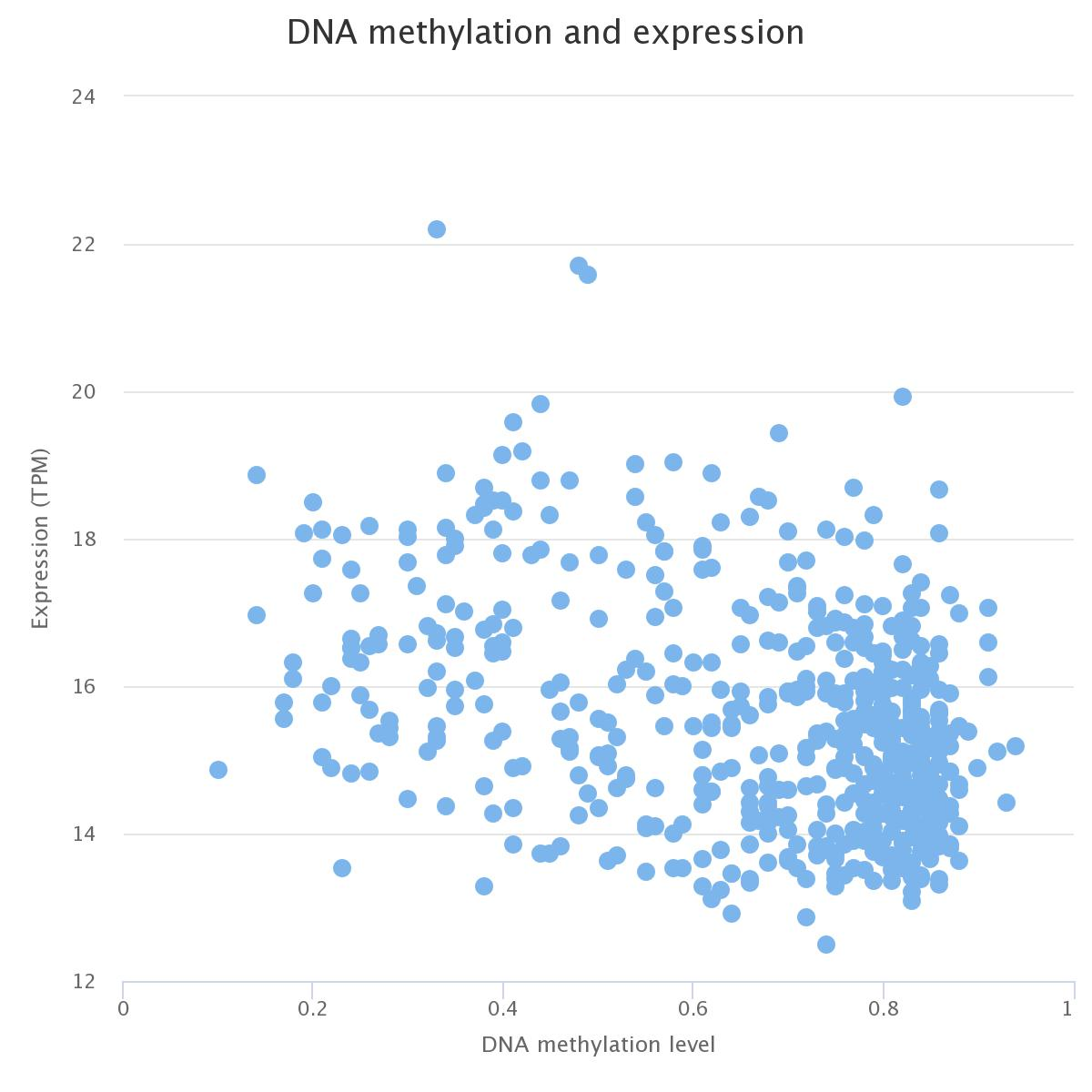

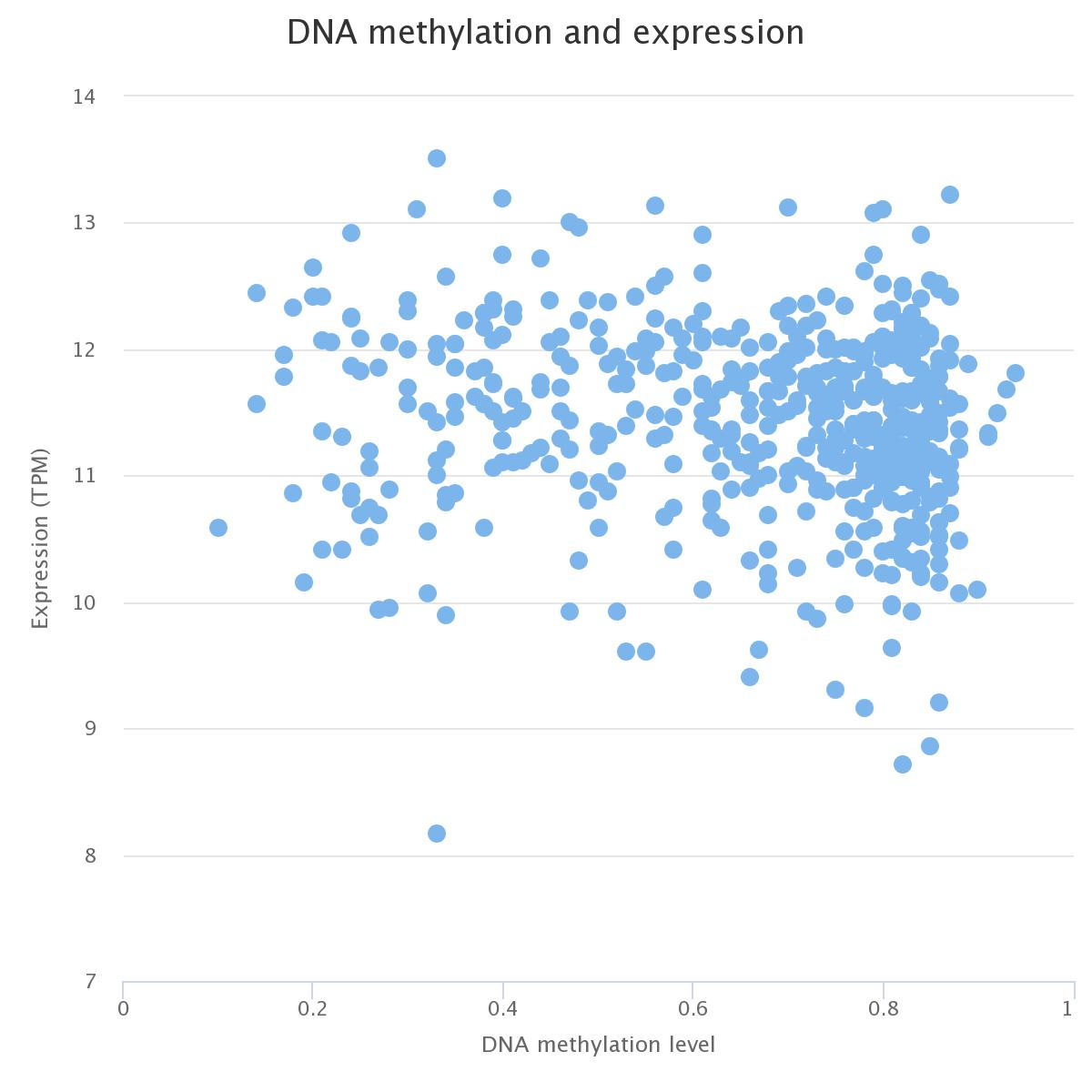


**k) CpG 6 - cg04028450 in Kidney**


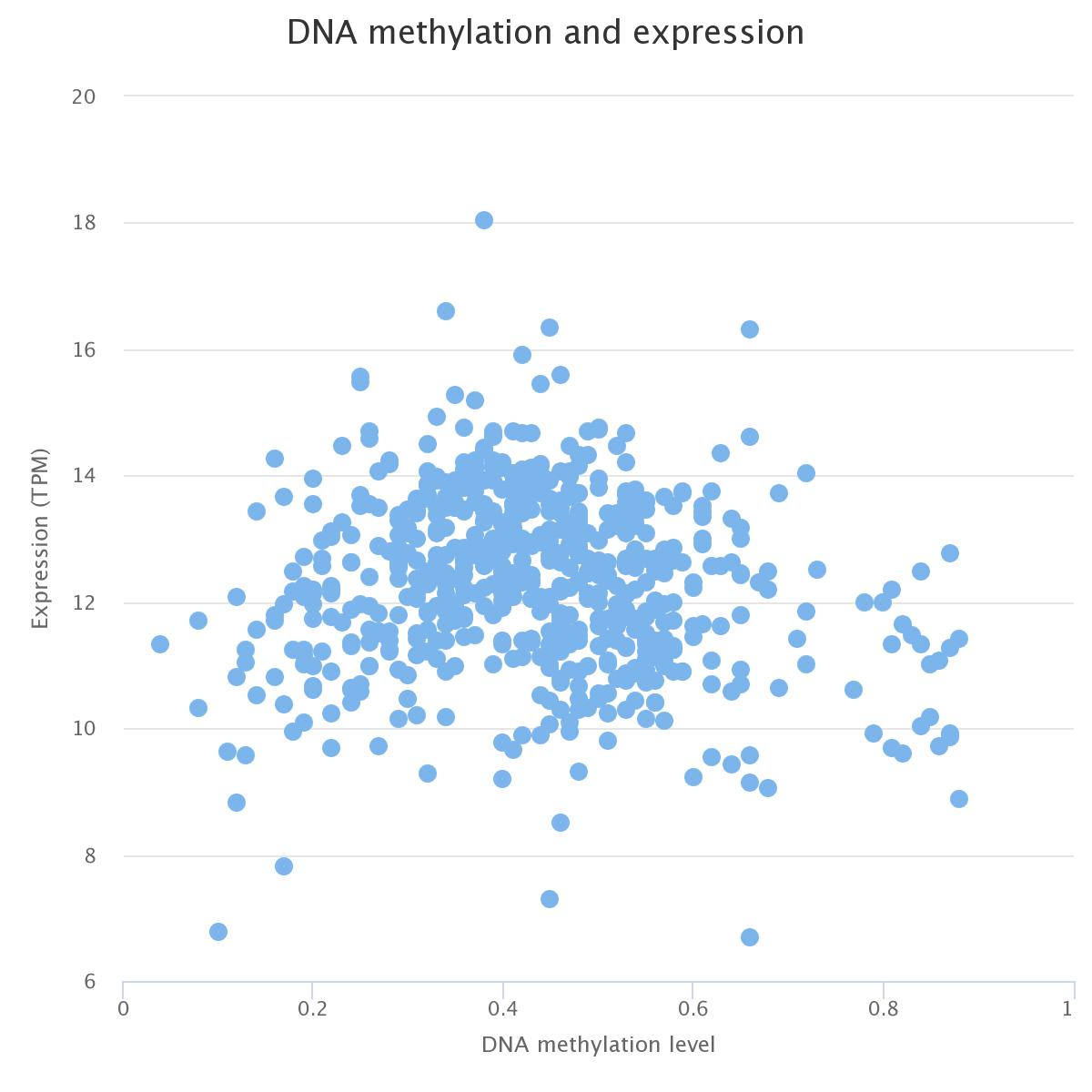


**Figure 6a-k:** Changes in the expression of the top 6 CpG sites. The x axes show the level of DNA methylation, while the Y axes show the expression levels in the specific tissues. Some sites exhibited changes in multiple tissues, while others, including CpG1 cg23604683 and CpG 3-cg21774457, exhibited no changes.

**T2DM Knowledge Graph**

**
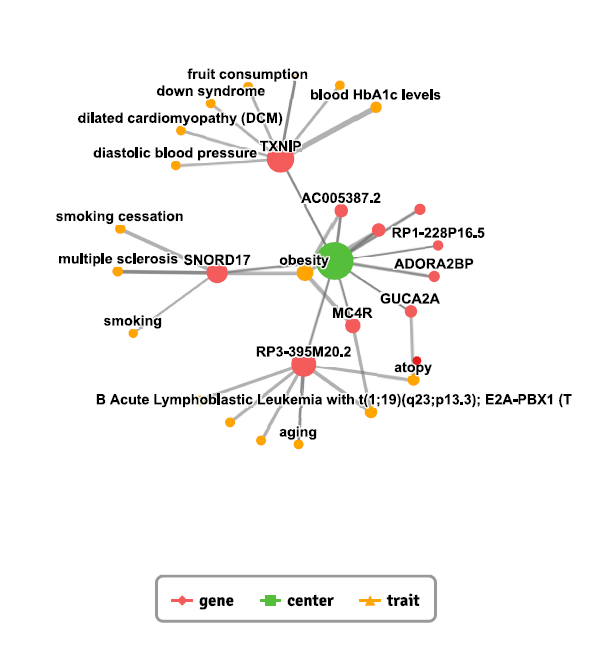
**

**Figure 7:** A network visualization of the relationship between T2DM and related phenotypes that have been previously reported. This graph is based on both previous publications and associations supported in the EWAS toolkit[90,91]. The center-colored green represents T2DM patients.

**Gene Ontology**


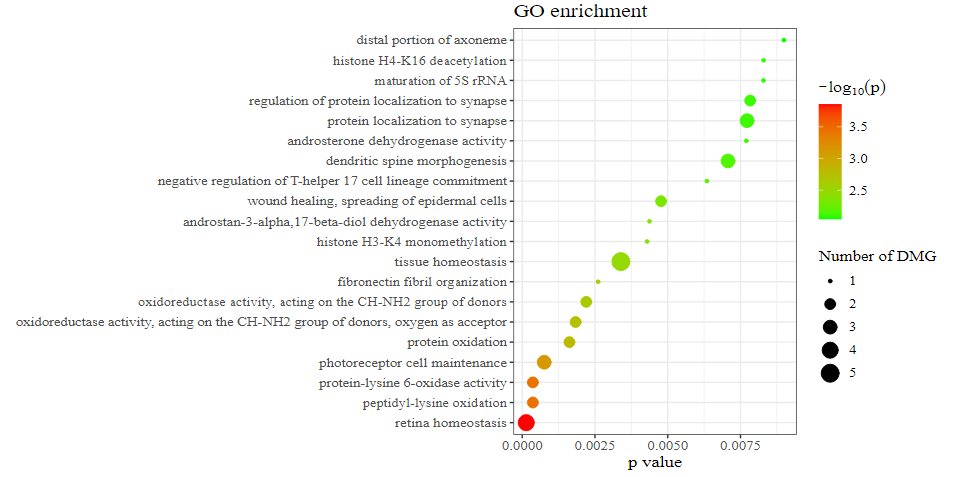


**Figure 8:** Enriched gene ontology terms of genes located close to the input CpG sites. Retina homeostasis was the most significantly enriched term.

**Chromatin state heatmap**

**Motif enrichment**

**Table 3:** Transcription factor name (DNA binding domain). This table shows the enriched motifs and their associated transcription factors among the top 100 DMPs (input probes).

| **Rank** | **Motif** | **Name** | **P Value** | **Log P - pvalue** | **Q -Value (Benjamini)** | **Target Sequences with Motif** | **Background Sequences with Motif** |
| --- | --- | --- | --- | --- | --- | --- | --- |
| **1** | 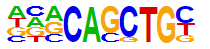 | E2A(bHLH)/proBcell-E2A-ChIP-Seq(GSE21978/Homer | 1e-2 | -5.082e+00 | 1.0000 | 30.0 (30%) | 90990.9 (19.18%) |
| **2** | 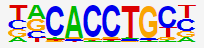 | Snail1(Zf)/LS174T-SNAIL1.HA-ChIP-Seq(GSE127183)/Homer | 1e-2 | -4.650e+00 | 1.0000 | 20.0 (20%) | 54597.6 (11.51%) |

**KEGG enrichment**


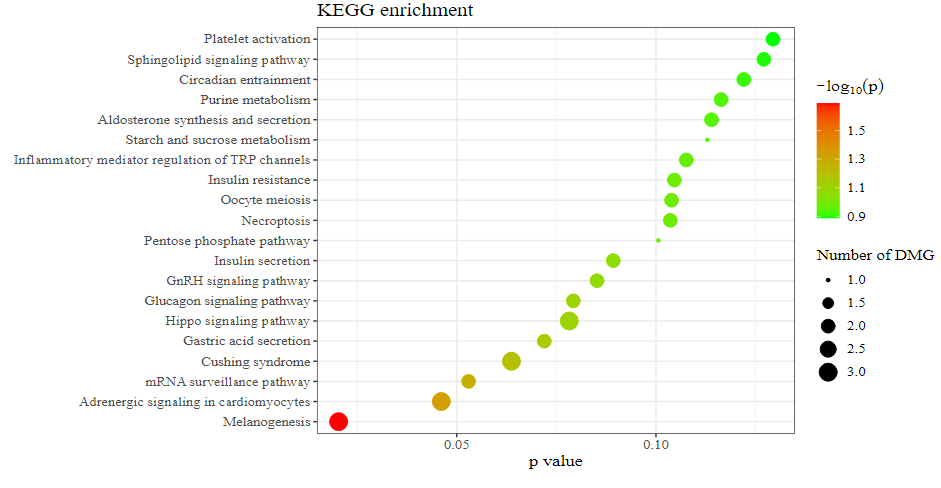


**Figure 9:** KEGG enrichment analysis of genes and genomes.
